# Conformational dynamics of the TTD–PHD histone reader module of the UHRF1 epigenetic regulator reveals multiple histone-binding states, allosteric regulation, and druggability

**DOI:** 10.1074/jbc.M117.799700

**Published:** 2017-10-26

**Authors:** R. Scott Houliston, Alexander Lemak, Aman Iqbal, Danton Ivanochko, Shili Duan, Lilia Kaustov, Michelle S. Ong, Lixin Fan, Guillermo Senisterra, Peter J. Brown, Yun-Xing Wang, Cheryl H. Arrowsmith

**Affiliations:** From the ‡ Princess Margaret Cancer Centre and Department of Medical Biophysics, University of Toronto, Toronto, Ontario M5G 1L7, Canada,; the § Structural Genomics Consortium, University of Toronto, Toronto, Ontario M5G 1L7, Canada,; the ¶ Small-Angle X-ray Scattering Core Facility, Frederick National Laboratory for Cancer Research, Leidos Biomedical Research Inc., Frederick, Maryland 21702, and; the ‖ NCI, National Institutes of Health, Frederick, Maryland 21702

**Keywords:** drug screening, epigenetics, molecular dynamics, molecular modeling, nuclear magnetic resonance (NMR), protein dynamic

## Abstract

UHRF1 is a key mediator of inheritance of epigenetic DNA methylation patterns during cell division and is a putative target for cancer therapy. Recent studies indicate that interdomain interactions critically influence UHRF1's chromatin-binding properties, including allosteric regulation of its histone binding. Here, using an integrative approach that combines small angle X-ray scattering, NMR spectroscopy, and molecular dynamics simulations, we characterized the dynamics of the tandem tudor domain–plant homeodomain (TTD–PHD) histone reader module, including its 20-residue interdomain linker. We found that the apo TTD–PHD module in solution comprises a dynamic ensemble of conformers, approximately half of which are compact conformations, with the linker lying in the TTD peptide–binding groove. These compact conformations are amenable to cooperative, high-affinity histone binding. In the remaining conformations, the linker position was in flux, and the reader adopted both extended and compact states. Using a small-molecule fragment screening approach, we identified a compound, 4-benzylpiperidine-1-carboximidamide, that binds to the TTD groove, competes with linker binding, and promotes open TTD–PHD conformations that are less efficient at H3K9me3 binding. Our work reveals a mechanism by which the dynamic TTD–PHD module can be allosterically targeted with small molecules to modulate its histone reader function for therapeutic or experimental purposes.

## Introduction

Epigenetic memory of cell identity requires the faithful propagation of DNA methylation patterns through cell division and is dependent on the function of UHRF1 (ubiquitin-like containing RING and PHD fingers 1). UHRF1 is a pentadomain protein that is targeted to hemimethylated DNA and repressive histone H3K9me3 modification states, where it recruits DNMT1 for the methylation of cytosine residues on daughter strands during DNA replication (for recent reviews of UHRF1, see Refs. 1–3). Dysregulation of DNA methylation is a hallmark of many cancers, and UHRF1 has been suggested as a target for anti-cancer therapy (2, 4). It is overexpressed in multiple cancer lines (5, 6) and thought to play a critical role in the down-regulation of tumor suppressor proteins (2). Importantly, the ability of UHRF1 to maintain DNA methylation patterns is dependent on its H3K9me3-binding function.

**Figure 1.**
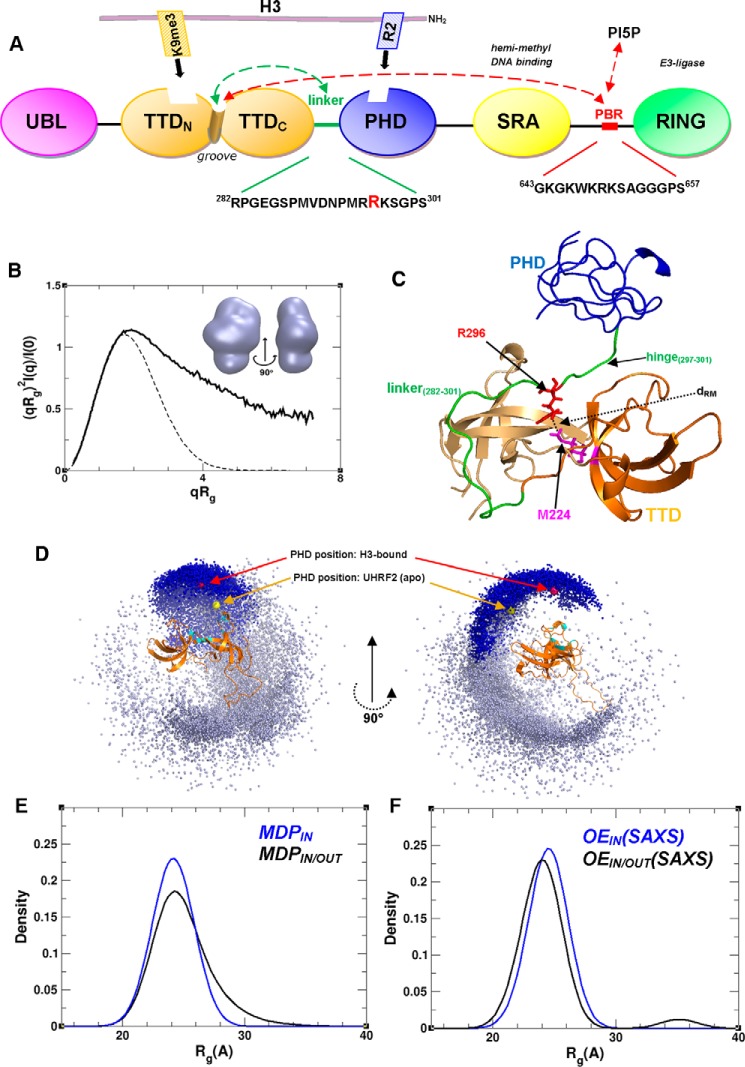
*A*, UHRF1 domain arrangement showing the basic function of each domain. The 20-residue TTD–PHD interdomain linker sequence is displayed (with the critical Arg^296^ in *red*), as well as that of the PBR, which has been implicated in allosteric regulation of histone binding through its reversible interaction with the TTD groove or PI5P (14). *B*, comparison of the experimental dimensionless Kratky plot for TTD–PHD (*solid line*) with the theoretical plot for a globular protein (*dashed line*). *Inset*, *ab initio* SAXS-predicted molecular envelope of the module is shown from two points of view. *C*, ribbon representation of TTD–PHD based on its structure in the H3-bound state (PDB code 3ASK) (10). The Cα atoms of Arg^296^ and Met^224^ are used as reference positions for the linker and groove, respectively, to assess their relative positioning (*d*_RM_) in various TTD–PHD conformers. *D*, the position of PHD centers of mass (calculated as the average position of the atoms in the PHD, weighted according to their mass) in TTD–PHD structures from MDP_IN_ (*dark blue spheres*) and MDP_IN/OUT_ (*dark* and *light blue spheres*) superimposed with the TTD (as a ribbon diagram). TTD residues that bind to the H3 peptide are displayed in *cyan*. The *red sphere* shows the PHD center of mass in the H3-bound UHRF1 TTD–PHD (PDB code 3ASK) (10). The *yellow sphere* shows the PHD center of mass in apo TTD–PHD of UHRF2 (crystal structure, PDB code 4TVR). *E*, *R*_g_ distribution in MDP_IN/OUT_ and MDP_IN_. *F*, *R*_g_ distribution in OE_IN/OUT_(SAXS) and OE_IN_(SAXS).

Hemimethylated DNA is recognized by the SRA domain (SET and RING-associated), whereas H3K9me3 marked chromatin is recognized by the plant homeodomain (PHD)^3^ and tandem tudor domain (TTD), which are connected by a 20-residue linker to form the TTD–PHD histone reader module (see Fig. 1*A*). The PHD in isolation recognizes the unmodified N terminus of H3 (7, 8), whereas the TTD binds to the H3K9me3 mark (9). The crystal structure of H3-bound TTD–PHD (10, 11) shows that the two domains can bind to their target sites cooperatively. This coordinate binding requires the linker to be bound to a peptide-binding groove formed at the interface of the TTD_N_/TTD_C_ subdomains; disruption of linker-groove contacts prevents high-affinity binding of TTD–PHD to H3K9me3. One putative mechanism to disrupt multivalent binding is by phosphorylation of the linker at Ser^298^ by PKA or PIM1 (10, 12, 13). It is unclear to what degree cooperative histone interaction occurs in the context of full-length UHRF1. Some studies indicate that the module may adopt histone-binding states that are solely PHD– or TTD–mediated (14, 15).

Histone and DNA binding by UHRF1 is regulated by long-range interdomain and linker-domain interactions within the full-length protein (14–17). Gelato *et al.* (14) showed that a polybasic region (PBR-UHRF1_643–657_) in the linker between the SRA and RING (really interesting and new gene) domains regulates the transition between PHD- and TTD–mediated histone reader states, through its reversible binding to the TTD groove or the phospholipid PI5P (see Fig. 1*A*). The PHD has also been reported to interact with the SRA domain in a UHRF1 state where histone binding is restricted (15, 16). These studies suggest that disruption of interdomain interactions could be a mechanism to pharmacologically target UHRF1. A detailed structural and dynamic picture of how these putative large-scale intramolecular rearrangements give rise to altered UHRF1-binding states remains elusive.

In this report we describe an integrated biophysical approach to characterize the scope of interdomain motion exhibited by the TTD–PHD histone reader module and its recognition of H3K9me3. In its apo form, we find that the unit is highly dynamic, populated with both extended and compact states. Using a fragment-based drug discovery approach, we identified a compound, 4-benzylpiperidine-1-carboximidamide (BPC) that binds to the open conformation, thereby preventing efficient recognition of H3K9me3.

## Results

### The UHRF1 TTD–PHD reader is highly dynamic in solution

To assess the conformational heterogeneity of the TTD–PHD histone reader module, we used small angle X-ray scattering (SAXS) in solution. The SAXS-derived *ab initio* molecular envelope is extended in shape with dimensions of ∼87 × 47 × 23 Å (Fig. 1*B*). The dimensionless Kratky plot is bell-shaped, with a maximum at a position that is shifted to higher coordinate values than expected for a globular protein and with poor convergence at high *q* values; this indicates the presence of flexibility/disorder. Furthermore, the average *R*_g_ value (24.5 Å) (Table 1) is more than 25% larger than the theoretical value expected for a globular protein of the same mass.

**Table 1:** **SAXS parameters for UHRF1 TTD–PHD with and without the presence of BPC**

SAXS parameters	TTD–PHD*^a^*	TTD–PHD/BPC*^b^*
*I*(0)*^c^*	0.057	0.021
*R*_g_ (Å)*^d^*	24.5	27.1
*R*_g_ (Å) real*^e^*	24.9	27.2
*D*_max_ (Å)*^f^*	83	94
*V*_c_*^g^*	274.3	284.4
*M*_w_*^h^*	25.5 (27.9)	24.2 (28.1)
NSD (SAXS envelope)*^i^*	0.75 ± 0.07	0.79 ± 0.02

*^a^* UHRF1_126–366_.

*^b^* UHRF1_126–366_ with BPC (4 mm, containing 4% DMSO).

*^c^* Intensity at *q* = 0.

*^d^* Based on Guinier fit.

*^e^* From GNOM (36).

*^f^* Maximum distance between atoms from GNOM.

*^g^* Volume of correlation (37).

*^h^ M*_w_ estimated from SAXS using *V*_c_ (37). The *M*_w_ expected from the sequence is shown in parentheses.

*^i^* NSD, normalized spatial discrepancy. The values are the average and standard deviation from 15 runs of DAMMIF (38).

In the published crystal structure of H3K9me3-bound TTD–PHD, the relative orientation of the two domains is fixed and stabilized by independent TTD and PHD interactions with the histone peptide and through the binding of the linker within the TTD groove (10), for which Arg^296^ is a key residue (Fig. 1*C*). Our SAXS analysis indicates that there is no specific orientation of the two domains relative to one another in the apo state. The level of interdomain motion within the reader module depends on conformational flexibility mediated by the linker and can be principally defined by two distinct types of mobility. In the first case, the Arg^296^-containing linker is bound within the TTD groove, and the mobility of the two domains is mediated largely by a 5-residue flexible “hinge” region (UHRF1_297–301_) (Fig. 1*C*). In the second case, the entire linker (UHRF1_282–301_) is flexible and exists in both TTD-bound and unbound states. The degree to which these two types of mobility are reflected in the dynamic behavior of the TTD–PHD unit was explored.

We employed an ensemble fitting approach in which molecular dynamics combined with rigid-body modeling was used to generate an initial set of conformations that approximate the conformational space available to the TTD–PHD reader. Then using the SES method (18), SAXS and NMR relaxation data were used to identify the dominant conformational states within the structural pool. We generated two initial sets of TTD–PHD conformations. The first set (molecular dynamics pool-IN (MDP_IN_)) contains 6,000 TTD–PHD conformations generated with the linker bound to the TTD groove. The second set (MDP_IN/OUT_) contains the entire MDP_IN_ pool and an additional 10,000 conformations with the linker displaced from the groove (Fig. 1*D*). SAXS data fitting was used to generate two optimal ensembles: OE_IN_(SAXS) and OE_IN/OUT_(SAXS), from their respective starting structural pools. Both ensembles fit the SAXS data equally well (χ_saxs_ = 0.21). The *R*_g_ distribution of OE_IN_(SAXS) has a broad peak centered at *R*_g_ = 24.5 Å, similar to its initial starting pool (MDP_IN_), whereas the OE_IN/OUT_(SAXS) displays a bimodal *R*_g_ distribution, with major (*R*_g_ = 24.0 Å) and minor (*R*_g_ = 35 Å) peaks corresponding to compact and extended reader conformations (Fig. 1, *E* and *F*). The SAXS-fitted OEs largely reproduce the conformational space of their starting pools, but in comparison to each other, do not overlap, as would be expected if the TTD–PHD unit possessed restricted flexibility (supplemental Fig. S1). Our fitting of the SAXS data suggests that OE_IN/OUT_(SAXS) reflects the dynamic range of TTD–PHD in solution more accurately than OE_IN_(SAXS) and provides evidence for TTD–PHD states where the groove is exposed.

### NMR relaxation data reflect partially coupled TTD–PHD interdomain motion

**Figure 2.**
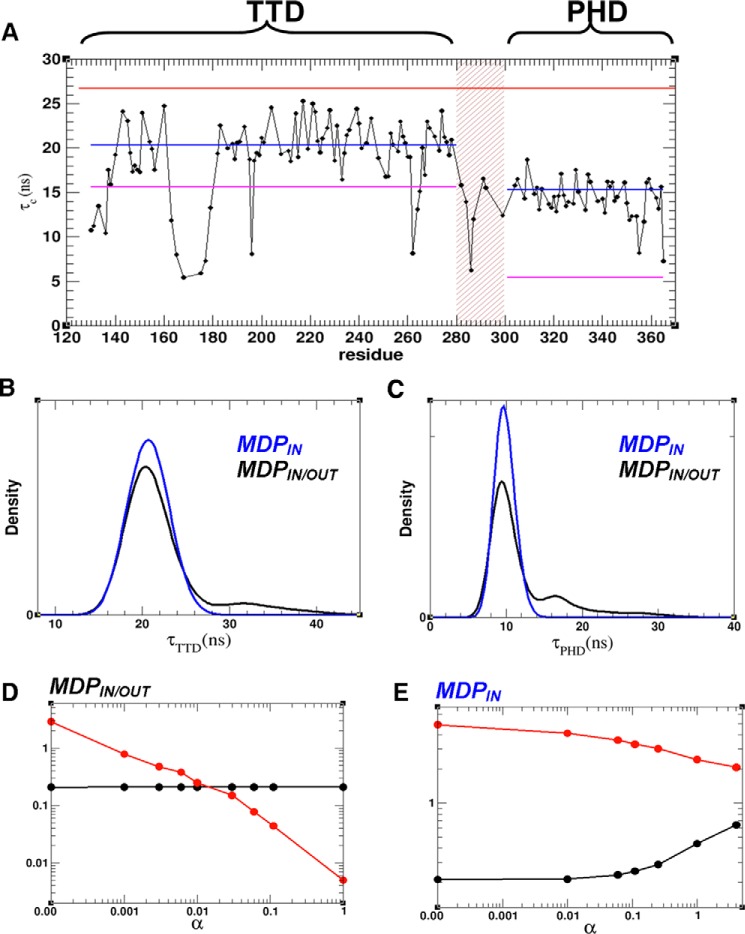
**Interdomain flexibility of the TTD–PHD module from NMR relaxation and SAXS data.** *A*, local correlation time (τ_c_) determined for each residue from experimental *T*_1_/*T*_2_ ratios (*black spheres*). The apparent global correlation times of the two domains derived by fitting to a standard diffusion model for each domain separately (*blue horizontal line*). The theoretical τ_c_ values predicted by HYDRONMR (20) for full-length TTD–PHD (*red horizontal line*) and for individual domains separately (*magenta horizontal line*). *B* and *C*, distribution of HYCUD-predicted (22) global τ_c_ values in MDP_IN/OUT_ and MDP_IN_ for the TTD (*B*) and PHD (*C*). *D* and *E*, goodness of fit of MDP_IN/OUT_ (*D*) and MDP_IN_ (*E*) to SAXS (χ_saxs_, *black circles*) and NMR (χ_relax_, *red circles*) data shown as a function of the weighting factor α. Ensemble fitting to both SAXS and NMR data were performed simultaneously by minimizing residual χ^2^ = χ_saxs_^2^ + α · χ_relax_^2^ at different values of α.

TROSY-based ^1^H/^15^N/^13^C triple-resonance backbone spectra were acquired to assign backbone TTD–PHD resonances (in a ∼80% deuterated sample). We could assign 203 amide resonances, of which 134 were located in the TTD, 11 in the linker and 58 in the PHD (supplemental Fig. S2). The assignments were used as the basis for residue-specific ^15^N relaxation and ^1^H-^15^N heteronuclear NOE measurements (supplemental Fig. S2 and supplemental Table S2). NOE values averaged across the ordered parts of the two domains are approximately the same (0.73 ± 0.06), with reduced values in loops (*e.g.* residues 163–180) and in the linker, indicating higher flexibility for these regions. The two domains exhibit different *T*_1_ and *T*_2_ values, and this is most evident comparing their local correlation time (τ_c_) determined for each residue (from *T*_1_/*T*_2_ ratios) (Fig. 2*A* and supplemental Table S2). The results of fitting NMR relaxation data to a standard diffusion model using the program MODELFREE (19) are shown in Table 2. The data fit better when the TTD and PHD are considered individually rather than as a single unit with the two domains rigidly attached (see reduced χ^2^ in Table 2), which indicates the presence of interdomain flexibility. The best fit for both domains was obtained with an axial symmetric diffusion model where τ_m_ = 20.32 ± 0.65 *ns* for the TTD, and τ_m_ = 15.36 ± 0.81 *ns* for the PHD (Table 2 and Fig. 2*A*). The domains are tumbling with different rates; however, the rotational correlation times values predicted by HYDRONMR (20) indicate that the domain motion is coupled (supplemental Table S3 and Fig. 2*A*).

**Table 2:** **Rotational diffusion parameters of the TTD and PHD within UHRF1 TTD–PHD** The diffusion parameters were determined by fitting the known crystal structure (PDB code 3ASK) to ^15^N-relaxation data (acquired at 800 MHz) using Modelfree4.15 (19). Fitting was performed for the full-length construct and each domain individually.

	TTD	PHD	TTD–PHD*^a^*
τ_m_ (ns)*^b^*	20.32 ± 0.65	15.36 ± 0.81	15.65 ± 0.07
Anisotropy*^c^*	1.17 ± 0.05	1.41 ± 0.06	1.37 ± 0.04
Reduced (*c*^2^)*^d^*	4.4	3.9	23.1

*^a^* UHRF1_126–366_.

*^b^* Overall rotational correlation time, τ_m_ = 1/2(*D*_par_ + 2*D*_per_), where *D*_par_ and *D*_per_ are parallel and perpendicular principal values of the axially symmetric rotational diffusion tensor, respectively.

*^c^* Degree of anisotropy of diffusion tensor, *D*_par_/*D*_2per_.

*^d^* Goodness of fit.

The HYCUD approach was recently developed to predict rotational correlation times of globular domains within flexible modular systems (21, 22). We used our two MD-generated pools of TTD–PHD structures (MDP_IN_ and MDP_IN/OUT_) to carry out HYCUD-based predictions of the effective rotational correlation times of the TTD (τ_TTD_) and PHD (τ_PHD_). Using MDP_IN_, both τ_PHD_ and τ_TTD_-predicted distributions are bell-shaped curves with peaks positioned at ∼ 10 and 21 ns, respectively (Fig. 2, *B* and *C*), whereas the distributions derived from predictions using MDP_IN/OUT_ are right-skewed with the major peaks extending up to ∼35 and ∼45 ns for τ_PHD_ and τ_TTD_, respectively. The predicted average τ_c_ values for the TTD from both MDP_IN/OUT_ and MDP_IN_ pools are in good agreement with the experimental value, but those for the PHD are smaller and in better agreement with the value predicted from MDP_IN/OUT_ (Tables 2 and 3 and supplemental Table S3).

**Table 3:** **Global structural parameters and goodness of fit to the experimental data for different ensembles of TTD–PHD**

Data used	OE_IN/OUT_*^a^*		MDP_IN/OUT_*^b^*	MDP_IN_*^b^*
	SAXS + NMR	SAXS		
χ_saxs_*^c^*	0.21	0.21	0.93	0.52
<τ_TTD_> (ns)*^d^*	20.9 ± 2.0	21.1 ± 1.7	22.0 ± 4.7	20.6 ± 1.9
<τ_PHD_> (ns)*^d^*	15.0 ± 8.1	9.9 ± 1.3	11.9 ± 4.9	9.7 ± 1.0
*R*_g_ (Å)*^e^*	24.1 ± 2.6	24.3 ± 2.4	24.8 ± 2.0	24.0 ± 0.9
*D*_max_ (Å)*^e^*	83.3 ± 8.4	85.6 ± 6.8	85.4 ± 7.9	85.8 ± 6.4
*P*_bound_*^f^* (%)	51	57	40	100

*^a^* OEs selected from MDP_IN/OUT_ using the SES method (18).

*^b^* MDP_IN/OUT_ and MDP_IN_ with uniform weights.

*^c^* Goodness of fit for fitting to SAXS data.

*^d^* Average and standard deviation values of the correlation time; τ_c_ for each member of the ensemble was predicted using the HYCUD method (21, 22).

*^e^* Average and standard deviation.

*^f^* Percentage of structures with the linker bound to the TTD groove.

### Ensemble fitting using both SAXS and NMR data indicates that the TTD–PHD module adopts compact and extended conformations

Using the HYCUD method, the correlation time (τ_c_) of a domain is calculated as a simple average from predicted τ_c_ values for all members of an initial structural ensemble, each with equal weighting. As we demonstrated above, the HYCUD-predicted τ_PHD_ and τ_TTD_ values do not agree with experimentally determined values. We therefore modified the HYCUD-based prediction algorithm by introducing non-uniform weights for conformers in the ensemble and then used the SES method to optimize and combine SAXS and NMR relaxation data to estimate appropriate weights. For this purpose, the discrepancy between the predicted and experimental data is measured χ^2^ = χ_saxs_^2^ + α · χ_relax_^2^, where χ_saxs_ and χ_relax_ measure the goodness of fit to SAXS and relaxation data, respectively (Equation 1), and the parameter α regulates the contribution of the relaxation data (see “Experimental procedures” for details). The results of SES fitting of the initial MDP_IN/OUT_ and MDP_IN_ pools at different values of the parameter α are shown in Fig. 2. In the case of MDP_IN/OUT_, increasing the value of α from 0 to 1 gradually results in progressive improvement of relaxation data fitting, whereas SAXS data fitting remains the same. At α = ∼0.01, an OE is generated from fitting MDP_IN/OUT_ to the SAXS and NMR data equally well (Fig. 2*D*). In contrast, we failed to find any value of α, such that MDP_IN_ can be fitted satisfactorily to both SAXS and NMR data (Fig. 2*E*).

**Figure 3.**
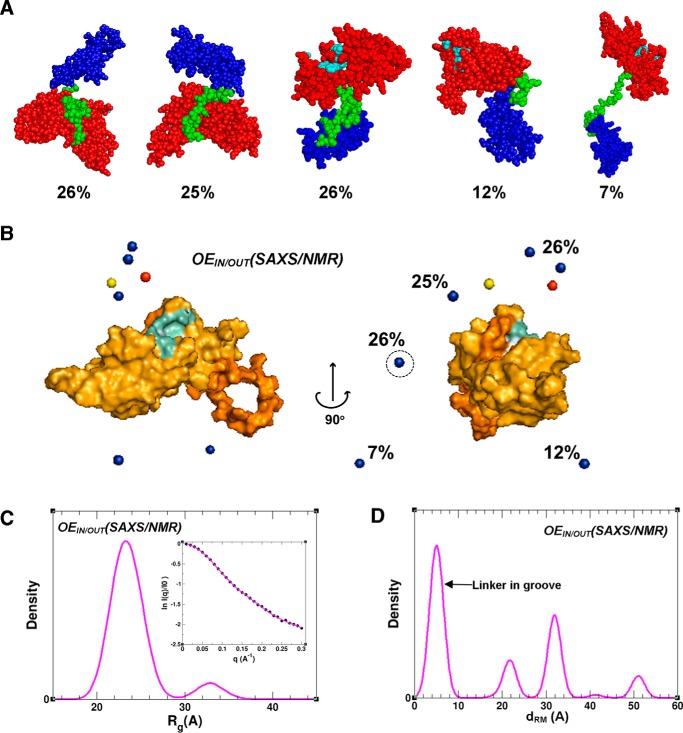
**Dynamic ensemble of the TTD–PHD reader in solution derived from SAXS and NMR data.** *A*, the most populated (based on weighted %) conformers from OE_IN/OUT_(SAXS/NMR). The TTD, PHD, and linker are colored in *red*, *blue*, and *green*, respectively. TTD residues that form the aromatic cage are displayed in *cyan. B*, the position of PHD centers of mass in the most populated conformers from OE_IN/OUT_(SAXS/NMR) (*blue spheres*) superimposed with the TTD (as a surface representation). Residues that bind to the H3 peptide are displayed in *cyan*. The *red sphere* shows the PHD center of mass in H3-bound UHRF1 TTD–PHD (PDB code 3ASK) (10), and the *yellow sphere* shows the PHD center of mass in apo TTD–PHD of UHRF2 (PDB code 4TVR). The average populations (%) are displayed. (See also supplemental Fig. S3 for comparison.) *C*, *R*_g_ distribution in OE_IN/OUT_(SAXS/NMR). The *inset* shows the experimental SAXS profile plotted with the theoretical profile averaged over the ensemble. *D*, the distribution of Cα-Cα distance between residues Arg^296^ and Met^224^ (*i.e. d*_RM_; refer to Fig. 1*C* diagram) in OE_IN/OUT_(SAXS/NMR).

We performed recovery of solution ensembles of the TTD–PHD by fitting MDP_IN/OUT_ to SAXS and NMR data (α = 0.06). The optimal ensemble, OE_IN/OUT_(SAXS/NMR), fits the SAXS data well (χ_saxs_ = 0.21), and likewise, the predicted τ_PHD_ (∼15.0 ns) and τ_TTD_ (∼20.9 ns) are in good agreement with experimental values (Tables 2 and 3). The five most populated states comprising 96% of this ensemble are shown in Fig. 3*A*. The structures can be divided into two groups based on the positioning of the linker. In the first group, which we call the “bound” state, the linker occupies the TTD groove. These conformers cluster around either the observed H3K9me3-bound TTD–PHD crystal structure, or interestingly, the domain arrangement seen in apo TTD–PHD of UHRF2 (Fig. 3*B* and supplemental Fig. S3). All conformers in the bound state are compact (Fig. 3, *C* and *D*), with an *R*_g_ of ∼22–24 Å, which corresponds to the major *R*_g_ distribution peak. In the second group, that we call the “open” state, the TTD groove is solvent-exposed. In this state, there are both compact and extended conformers that correspond to the major *R*_g_ distribution peak (∼24 Å) and to the minor peak centered at ∼33 Å (Fig. 3*C*). The relative population of bound states in OE_IN/OUT_(SAXS/NMR) is 51% (Table 3).

### Allosteric modulation of the TTD–PHD module with small molecules

There is strong evidence that the 15-residue PBR (UHRF1_643–657_) between the SRA and RING domains is involved in the allosteric regulation of TTD–PHD histone binding. This occurs through competitive displacement of the linker from the TTD groove (Fig. 1*A*). In full-length UHRF1, this results in the failure of UHRF1 to recognize H3K9me3 caused by a transition from TTD–mediated to PHD–mediated histone binding (14). We performed a screen of fragment-sized small molecules to identify compounds that could, in a manner analogous to the PBR, bind to the TTD groove, block linker–groove interactions, and promote open TTD–PHD conformations.

**Figure 4.**
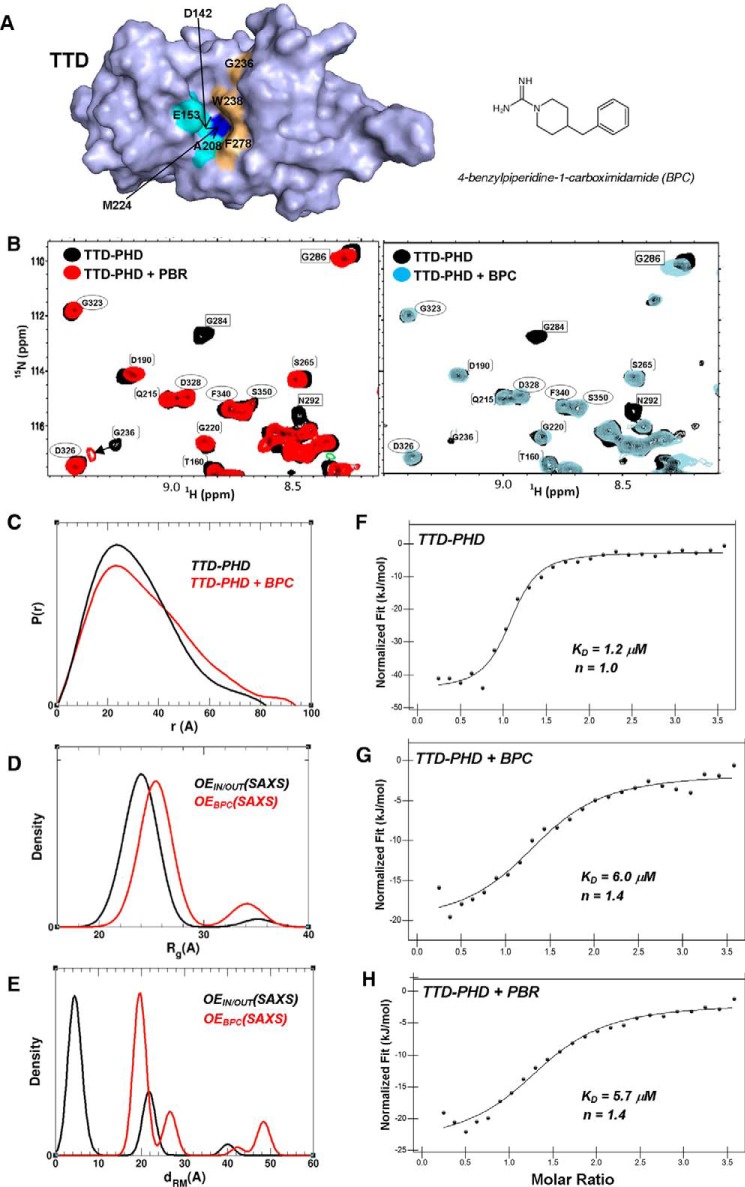
**BPC and PBR peptide binding promote open TTD–PHD conformations.** *A*, BPC (with its chemical structure displayed) binds close to the Arg^296^-binding pocket. Asp^142^, Glu^153^, Ala^208^, Met^224^, Trp^238^, and Phe^278^ combine to form the pocket in the TTD groove. Residues showing significant chemical shift perturbations upon BPC binding are colored *orange*, and those that are not assigned in this construct are in *cyan*. Met^224^ (*blue*), at the base of the pocket, is not significantly shifted. *B*, (^1^H-^15^N) TROSY overlays show that titration of TTD–PHD with either BPC or PBR peptide (at ∼250 μm protein/750 μm ligand) show substantial broadening and/or movement of linker resonances (*boxed*). Some TTD residues (*brackets*) at the interface of the groove (*e.g.* Gly^236^) are shifted or broadened as well. PHD resonances remain largely unaffected by binding (*ovals*). For clarity, only well resolved resonances are labeled. *C*, normalized pair distance distribution function *P*(*r*) calculated from SAXS data for TTD–PHD bound to BPC (4 mm, 4% DMSO) and for apo TTD–PHD. *D*, comparison of the *R*_g_ distribution in OE_IN/OUT_(SAXS) and OE_BPC_(SAXS) derived from SAXS data for apo and BPC-bound TTD–PHD, respectively. *E*, distribution of d_RM_ (refer to Fig. 1*C* diagram) in the OE_IN/OUT_(SAXS) and OE_BPC_(SAXS). *F*, ITC curves show a cooperative profile for TTD–PHD binding to H3K9me3_(1–15)_. *G* and *H*, the presence of BPC (at 1.5 mm, 4% DMSO, ∼ 30:1 fragment:protein) (*G*) or PBR peptide (at 1.5 mm, ∼30:1 peptide:protein) (*H*) reduces the TTD–PHD binding affinity for the histone peptide. This is consistent with a putative shift from a cooperative to a PHD–mediated binding mode.

We designed a fragment library containing 2,040 compounds that was initially screened against isolated TTD (UHRF1_121–286_) using a fluorescence polarization (FP) assay that tracked the displacement of a N-terminally tagged H3K9me3 peptide (H3K9me3_(1–25)_). From this screen, eight putative TTD-binding hits were identified. Analysis of amide peak movement in the (^1^H-^15^N) HSQC spectra of the TTD in the presence of the fragments indicates that the binding of two, BPC and a tricyclic imine, occurs in the groove. Because of limited commercial availability of the tricyclic amine, we focused only on BPC. The binding of this compound occurs close to (or within) the Arg^296^-binding pocket; we observe significant perturbation of Trp^238^ and Phe^278^ resonances that form part of the pocket, and in Gly^236^, which is directly adjacent to it (Fig. 4*A* and supplemental Fig. S5). We also see chemical shift changes in non-surface exposed residues that are close to or are in contact with the groove. A similar amide peak perturbation pattern is observed when the TTD is titrated with a 15-residue peptide corresponding to the PBR, resulting from its binding in the groove (supplemental Fig. S5). Further characterization of BPC binding to isolated TTD was performed using ITC and DSF, with an estimated *K_D_* of 50 μm (supplemental Fig. S5) and a calculated ligand efficiency of 0.38 (which is defined as the binding energy per heavy atom).

We used NMR and SAXS analysis to assess whether BPC could promote open conformations of TTD–PHD, in a manner comparable with that of the UHRF1 PBR. In (^1^H-^15^N) TROSY spectra of the reader titrated with BPC, significant conformational broadening and/or movement of amide peaks can be observed in residues spanning the entire length of the linker. Exchange broadening is clearly observed for Arg^282^, Gly^284^, Asp^291^, Asn^292^, Met^294^, and Ser^301^ amide resonances at 1:3 protein/BPC ratios, with perturbations also seen with Gly^286^ and Gly^299^ (Fig. 4 and supplemental Fig. S6). TTD residues that form part of or are close to the Arg^296^-binding pocket (*e.g.* the ^236^GFW^238^ triad and Ala^208^) also exhibit exchange broadening and/or chemical shift changes, consistent with compound binding close to this site (supplemental Fig. S6). PHD resonances are, by comparison, unaffected by the presence of BPC, and in a separate titration, we confirmed there was no interaction between the fragment and this domain (supplemental Fig. S8). A strikingly similar peak broadening/perturbation pattern is observed in spectra of TTD–PHD when it is titrated with the 15-residue PBR peptide (Fig. 4*B* and supplemental Fig. S6).

SAXS profiles of TTD–PHD collected in the presence of BPC demonstrate an increase in the overall dimensions resulting from BPC binding (Tables 1, supplemental Table S1, and Fig. 4*C*). The *R*_g_-based Kratky plot is shifted to higher coordinate values with respect to its position for apo TTD–PHD (supplemental Fig. S4). Also, the pair-distance distribution function exhibits a broad extended tail with a shallow secondary shoulder observed at ∼50 Å (Fig. 4*C*), indicating extended reader conformations. We performed SES fitting of SAXS data collected for TTD–PHD in the presence of BPC using MDP_IN/OUT_. The optimal ensemble, OE_BPC_(SAXS), fits the data reasonably well in the *q* range of 0 < *q* < 0.2. The *R*_g_ distribution is bimodal with the major peak at ∼25 Å and a minor peak at ∼34 Å (Fig. 4*D*). In comparison with the *R*_g_ distribution of the optimal ensemble generated for apo TTD–PHD (*i.e.* OE_IN/OUT_(SAXS)), the position of the major peak is shifted by 1 Å, and the minor peak has a larger height, indicating that there is a higher percentage of extended conformations. Interestingly, the distance that specifies the relative position of the linker with respect to the TTD groove (*d*_RM_) shows that the 6,000 starting structures in the pool in which the linker is bound to the groove make zero contribution to OE_BPC_(SAXS) (Fig. 4*E*). Taken together, these data indicate that BPC binds in the TTD groove and disrupts the interaction with the linker, shifting the ensemble equilibrium toward less compact structures. These less-compact, “linker-out” conformers should be unable to bind to H3K9me3 peptides in a cooperative manner (as in PDB code 3ASK (10, 23)) and, in addition, be unable to bind via the isolated TTD because groove-H3K9me3 contacts found to be essential for this binding mode, as reported by Nady *et al.* (9), are blocked.

To evaluate the effect of BPC on histone binding by the reader module, we compared the binding affinity of TTD–PHD with H3K9me3 peptide in the presence of both BPC and PBR peptide (Fig. 4). Apo TTD–PHD was found to exhibit cooperative H3K9me3_(1–15)_ binding (*K_D_* = 1.2 μm, *n* = 1.0) resulting from dual TTD-/PHD–mediated interaction, consistent with previous studies (10) (Fig. 4*F* and supplemental Fig. S9). The increased *K_D_* (∼5-fold) for H3K9me3 in the presence of BPC (Fig. 4*G*) or PBR peptide (Fig. 4*H*) is fully consistent with a transition from a cooperative (*i.e.* PHD– and TTD–dependent) binding mode to one mediated by the PHD only.

## Discussion

Large-scale intramolecular rearrangements play a critical role in UHRF1 function, consistent with a dynamic framework in which its conformational equilibria are shifted in response to the chromatin state and aggregate presence of other proteins and cellular factors with modulating influences (14–16). One noted example of this conformational modulation is that induced by the lipid PI5P, which regulates the transition between TTD- and PHD–mediated histone-binding states through its reversible interaction with the PBR element (UHRF1_643–657_) (14) (Fig. 1*A*). Structural insight into large-scale UHRF1 intramolecular rearrangements has been obscure.

**Figure 5.**
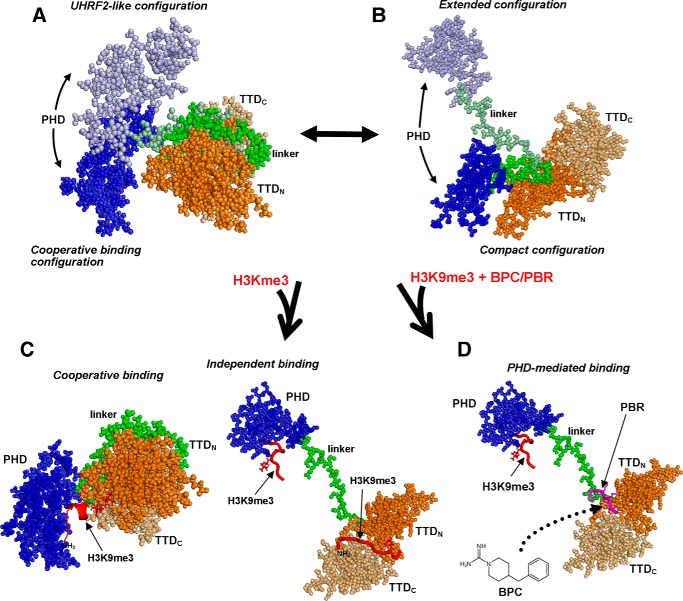
**Apo and H3-binding states of the TTD–PHD histone reader.** The apo TTD–PHD OE is consistent with conformers in which the linker is positioned both in (*A*) and out (*B*) of the TTD groove. When the linker is in the groove, the relative positions of the domains have two favorable orientations: the first is very similar to the H3-bound state described by Arita *et al.* (10) where histone binding is cooperatively mediated by the PHD and TTD (*C*); the second resembles the orientation adopted by apo TTD–PHD in its paralog UHRF2. Both extended and compact conformers are populated when the linker is out of the groove (*B*) and provide a clear mechanism for histone-binding states mediated independently by the PHD and/or TTD (*C*). Gelato *et al.* (14) described the formation of a PBR-bound state where the linker is forced out of the groove as a mechanism for allosteric regulation of histone binding; BPC can similarly promote open reader states (*D*).

Our study shows that the UHRF1 TTD–PHD histone reader is highly dynamic in its apo state, with clear evidence of open and extended conformations (Fig. 5). We have applied a novel integrated approach where both SAXS and NMR relaxation data are used to determine OEs from starting pools of conformers derived from MD simulations and rigid-body modeling. Our results indicate that TTD–PHD structures in which the linker is bound to the TTD groove are not favored over those where it is unbound. Linker positioning has been shown to be a critical determinant of histone-binding behavior, because cooperative, high-affinity interaction necessitates that it occupy the groove (10). Interestingly, even when the linker is positioned in the groove, there is evidence of additional flexibility mediated by the hinge region (UHRF1_297–301_). The linker-bound structures (Fig. 5*A*) in our OE are clustered around two configurations: in the first, the domain orientation is similar to that seen in the cooperative binding mode (PDB code 3ASK), whereas in the second, the domain orientation is similar to that seen in the crystal structure of apo UHRF2 TTD–PHD (PDB code 4TVR). Open conformers where the linker is out of the groove (Fig. 5*B*) reveal a vulnerability of the TTD to allosteric regulation by competitive binding of entities such as the PBR or drug-like small molecules (Fig. 5*D*).

UHRF1 is a potential therapeutic target, because it is essential for the maintenance of DNA methylation patterns and highly expressed in most cancers (2, 4). To date, only one UHRF1 inhibitor has been identified, a uracil derivative that interferes with the SRA domain (24). Because H3K9me3 binding is a requirement for UHRF1 function (25) and can be disrupted by the PBR through its association with the TTD (14), we hypothesized that allosteric disruption of the cooperative (23), high-affinity binding mode of the TTD–PHD module may be an attractive strategy for small molecule antagonists of UHRF1 function. An important aspect of intramolecular interactions is the high effective concentration of interacting regions within a single macromolecule. Such effects may pose a challenge for the development of drug-like small molecules that can efficiently compete with these interactions. Here, using an FP H3-peptide displacement assay with the isolated TTD, we identified the small fragment BPC as a linker-competitive binder to the TTD groove, with a *K_D_* close to 50 μm (supplemental Figs. S5 and S8). Saturating concentrations of this highly soluble compound can induce open conformers of the TTD–PHD module and reduce its affinity for H3K9me3 peptides (Fig. 4 and supplemental Figs. S5 and S6). BPC should prove useful as a tool for *in vitro* investigations that seek to relate open TTD–PHD conformations with specific UHRF1-binding modes. The small size and high ligand efficiency of the compound suggest that it can be further optimized for potency. An attractive approach could be to link compounds such as BPC that bind in the TTD groove, with those designed for interaction with the aromatic cage, which recognizes the trimethyl lysine of H3K9me3. Recent successes in identifying small molecule antagonists of methyl lysine reader domains bodes well for this approach (26–28).

## Experimental procedures

### Protein expression and purification

UHRF1_126–366_ corresponding to the TTD–PHD module and UHRF1_121–286_ corresponding to the TTD were expressed and purified as described previously (9). TTD–PHD was purified with and without an N-terminal His tag, and in cases where the tag was removed, this was by overnight incubation with TEV protease at 4 °C (9). For isotopically labeled proteins used for NMR spectroscopy (^15^N-labeled, ^15^N/^13^C-labeled, or ^15^N/^13^C/^2^H-labeled), cells were grown in M9 minimal medium supplemented with [^15^N]ammonium chloride (1 g/liter), [^13^C]glucose (2 g/liter) when required, and 80% D_2_O when required.

### NMR spectroscopy

NMR samples were buffered in 20 mm sodium phosphate (pH 7.5), 150 mm NaCl, 5 mm DTT, 5 mm β-mercaptoethanol, 2 mm TCEP, and 10 μm ZnSO_4_. The protein concentration was between 200 and 300 μm for all samples. The data were collected at 25 °C on Bruker spectrometers equipped with cryoprobes and operating at 500, 600, or 800 MHz. The assignment of backbone TTD–PHD resonances was accomplished using the ABACUS method (29) for which standard backbone and ^15^N-edited NOESY spectra were collected using ^15^N/^13^C/^2^H- or ^15^N/^13^C-labeled protein. All 3D experiments were acquired using non-uniform sampling and processed using the software MDDGUI (30) or qMDD (31). ^15^N-Labeled TTD–PHD was used to acquire ^15^N *T*_1_ and *T*_2_ relaxation and ^1^H-^15^N heteronuclear NOE measurements (32) collected using standard Bruker pulse schemes in an interleaved manner. For *T*_1_ measurements, the variable delay was set to 0.1, 0.4, 0.8, 1.5, 2.0, 2.5, 3.5, and 5 s. For *T*_2_ measurements, the variable delay was set to 16, 32, 48, 64, 80, 96, 128, and 144 ms. The D1 was 3 s for all experiments. Reported values were the average from two measurements. All spectra were processed using NMRPipe (33) and analyzed with SPARKY (34).

### SAXS data collection and analysis

All SAXS samples were buffered in 20 mm Tris (pH 7.5), 150 mm NaCl, 5 mm DTT, 5 mm β-mercaptoethanol, 2 mm TCEP and 10 μm ZnCl_2_, and data were collected at concentrations ranging from ∼1 to 5 mg/ml. Measurements were carried out at Beamline 12-ID-B of the Advanced Photon Source, Argonne National Laboratory. The energy of the X-ray beam was 14 Kev (wavelength λ = 0.8856 Å), and two setups (small- and wide-angle X-ray scattering) were used simultaneously in which the sample to 2M detector distance was adjusted to achieve scattering *q* values of 0.006 < *q* < 2.6 Å^−1^, where *q* = (4π/λ)sinθ, and 2θ is the scattering angle. To reduce radiation damage and obtain good statistics, thirty 2D images were recorded for each buffer or sample solution using a flow cell, with an accumulated exposure time of 0.4–2.0 s. No radiation damage was observed as confirmed by the absence of systematic signal changes in sequentially collected X-ray scattering images. The scattering profile of the protein was calculated by subtracting the background buffer contribution from the sample buffer profile using the program PRIMUS (ATSAS package, EMBL) (35). Concentration series measurements for a sample were carried out to remove the scattering contribution caused by interparticle interactions and to extrapolate the data to infinite dilution. Guinier analysis and the experimental radius of gyration (*R*_g_) estimation from the data of infinite dilution were performed using PRIMUS. The pair distance distribution function, *P*(*r*), and the maximum dimension of the protein, *D*_max_, in real space were calculated with the indirect Fourier transform using the program GNOM (36). To avoid underestimation of the molecular dimension and consequent distortion in low resolution structural reconstruction, the parameter *D*_max_, the upper end of distance *r*, was chosen such that the resulting PDDF has a short, near zero-value tail at large *r*. The *R*_g_ from *P*(*r*) analysis was also reported. The molecular weights were estimated using *V*_c_ (37) in the *q* range of 0 < *q* < 0.3 Å^−1^. Fifteen *ab initio* shape reconstructions (molecular envelopes) were generated using DAMMIF (38) and averaged with DAMAVER (39). The structural models were superimposed and overlaid with the averaged envelope using SUPCOMP (40). The theoretical scattering intensity of a structural model was calculated and fitted to the experimental scattering intensity using CRYSOL (41).

### Structural characterization of TTD–PHD using SAXS and NMR data

We used an ensemble approach for the structural characterization of the TTD–PHD in solution by utilizing the SES protocol (18). The strategy on which the SES method is based consists of two main steps: 1) generate the initial ensemble of conformations to approximate the conformational space available for a system in solution and 2) find the optimal population weight for each member of the initial ensemble that minimizes the discrepancy between the ensemble-predicted and the observed experimental data. The goodness of the ensemble fit is measured as χ^2^. We used the following expression for χ^2^,

(Eq. 1) $$\chi^{2}=\chi_{\text{saxs}}^{2}+\alpha \;\text{·}\;\chi_{\text{relax}}^{2}$$

where

(Eq. 2) $$\chi_{\text{saxs}}^{2}={\sum_{i=1}^{N_{\text{q}}}\left[\frac{\sum_{k=1}^{{\text{N}}_{\text{ens}}}I_{\text{calc}}^{\text{k}}\left(q_{\text{i}}\right)\text{·}w_{\text{k}}-I_{\exp}\left(q_{\text{i}}\right)}{\sigma_{\text{saxs}}\left(q_{\text{i}}\right)}\right]}^{2}$$

(Eq. 3) $$\chi_{\text{relax}}^{2}={\left(\sum_{k=1}^{N_{\text{ens}}}\tau_{\text{TTD}}^{\text{k}}\text{·}w_{\text{k}}-\tau_{\text{TTD}}^{\text{exp}}\right)}^{2}/\sigma_{TTD}^{2}+{\left(\sum_{k=1}^{N_{\text{ens}}}\tau_{\text{PHD}}^{\text{k}}\text{·}w_{\text{k}}-\tau_{\text{PHD}}^{\text{exp}}\right)}^{2}/\sigma_{PHD}^{2}$$

where *I*_exp_(*q*) is the experimental SAXS scattering intensity, *N*_q_ is the number of experimental points, σ_saxs_(*q*) is the experimental error, I_calc_^*k*^ is the scattering intensity predicted for the *k*th conformation, τ_TTD_^exp^ and τ_PHD_^exp^ are the experimental rotational correlation times for the TTD and PHD, respectively, σ_TTD_ and σ_PHD_ are the experimental errors, τ_TTD_^*k*^ and τ_PHD_^*k*^ are correlation times predicted for the *k*th conformation, *N*_ens_ is the number of conformations in the initial ensemble, *w*_k_ is the population weight associated with the *k*th conformation in the ensemble, and α is the weighting factor for the NMR relaxation data. In the case α = 0, only SAXS data is used to optimize weights *w*_k_. Equation 1 can be represented in the matrix form,

(Eq. 4) $$\chi^{2}\left(w,\alpha \right)={\left\|C\left(\alpha \right)\cdot w-B\right\|}_{2}^{2}$$

where matrix C is of size (*N*_q_ + 2, *N*_ens_) and consists of predicted SAXS and NMR relaxation data for all members of the initial ensemble, matrix B consists of corresponding experimental values, ||·|| is the vector *l*_2_-norm, and ***w*** is the vector of weights. To construct the C matrix, the 30-point SAXS profiles in the range 0 < *q* < 0.3 Å^−1^ were predicted using CRYSOL, and two overall rotational correlation times, one for the TTD and one for the PHD, were predicted using HYCUD (21, 22). The ill-posed problem of finding vector of weights, ***w***, that minimizes χ^2^(***w***, α) under the condition ***w***_k_ ≥ 0 is solved using the SES approach (18). We used the SES module of the ARMOR package to generate a number of sparse solutions for different ensemble sizes with multiorthogonal matching pursuit algorithm. The ARMOR output was analyzed to select the optimal ensemble size using the *l*-curve (18), and the vector of the optimal weights, ***w***, was calculated by averaging over top near optimal solutions with similar value of χ^2^.

### RBP_IN/OUT_ and RBP_IN_ ensemble generation

Rigid-body pools were generated using RANCH (42). In these simulations, ordered parts of the TTD–PHD were assumed to be rigid, whereas disordered parts were represented by random chains. RBP_IN/OUT_ was generated by assuming that the His tag (in constructs containing it), the flexible TDD_N_ loop (UHRF1_163–180_), and the entire linker (UHRF1_282–301_) are disordered. RBP_IN_ was generated assuming that the His tag (for constructs containing it), the flexible TDD_N_ loop (UHRF1_163–180_), and the five-residue hinge region of the linker (UHRF1_297–301_) are disordered. Both pools consist of 30,000 TTD–PHD conformations.

### MDP_IN/OUT_ and MDP_IN_ generation

The initial pools of TTD–PHD conformations were obtained in two steps. In the first step, we performed all-atom MD simulations of TTD–PHD. Eight replica MD trajectories, each ∼150 ns long, were generated at 300 K. Four of these replicas were started from different conformations with the TTD groove occupied by the linker, whereas the other four replicas were started from conformations with the linker displaced from the groove. No bound-to-unbound or unbound-to-bound transitions of the linker were observed along the MD trajectories. Over the course of the simulations, the two domains retained their overall structure, with no ordered-to-disordered transitions, as indicated by the low root mean square deviation for TTD and PHD backbone atoms (∼3.5 and 3.3 Å, respectively) in MD-generated conformers. This is in agreement with NMR data (supplemental Fig. S7). Each 10-ps frame was saved during MD simulations, which resulted in 65,200 conformations of the TTD–PHD with the linker positioned in the groove and 55,460 conformations with the linker out of the groove. In the second step, we performed *k*-means clustering of the generated conformations using metrics that specify the relative position of the TTD and PHD, yielding 6,000 and 8,000 clusters of conformers with the linker in and out of the groove, respectively. To improve on relatively poor sampling of the conformers with the linker out of the groove in MD simulations, we also added 2,000 additional clusters, produced initially by rigid-body modeling, of open/extended TTD–PHD conformers. The representative structures of these clusters were used to construct MDP_IN/OUT_ and MDP_IN_.

### All-atom molecular dynamics simulations

A modified Generalized Born implicit solvent model (43) was exploited in the MD simulations to accelerate sampling of the conformational space for each of the systems. All simulations used an integration step of 2 fs with fixed bonds between hydrogen atoms and heavy atoms. The temperature was controlled by carrying out Langevin dynamics with the damping coefficient set to 2 or 5 ps^−1^. The cut-off for non-bonded Lennard-Jones and electrostatic interactions was set to 18 Å. The ionic strength was set to 0.15 m. All simulations were performed using NAMD 2.9 code (44) with the AMBER Parm99SB parameter set (45). A zinc AMBER Force Field (46) was used for PHD residues that coordinate three zinc ions.

### FP, ITC, and DSF measurements

For FP assays, the TTD (UHRF1_121–286_) was buffered in 20 mm Tris (pH 8.5), 50 mm NaCl, 3% DMSO, and 0.01% TX100. N-terminal FITC-labeled H3K9me3_(1–25)_ peptide was synthesized and purified by Tufts University Core Services (Boston, MA). Titrations and compound binding assays were performed in a 10-μl volume at a constant labeled peptide concentration of 0.04 μm. For compound screening and titrations, a non-saturating fixed protein concentration of 8 μm TTD was used. FP assays were performed in 384-well Axygen plates using a Synergy 4 microplate reader (BioTek). An excitation wavelength of 485 nm and an emission wavelength of 528 nm were used.

For ITC measurements of TTD–PHD (UHRF1_126–366_) interaction with H3K9me3, samples were dialyzed into a buffer containing 10 mm HEPES (pH 7.5), 150 mm NaCl, 1 mm TCEP, and 30 μm ZnCl_2_. H3K9me3_(1–15)_ peptide, in the same buffer, was brought to a concentration of 0.50 mm. A preliminary peptide injection of 0.06 μl was followed by subsequent 2-μl injections into the sample cell containing 167 μl of 50 μm TTD–PHD. Where indicated, 1.5 mm BPC or 1.5 mm PBR peptide was included in the injection syringe and sample cell. The reported *K_D_* and *n* values are based on the average from two measurements. For ITC measurements of TTD (UHRF1_121–286_) interaction with BPC, the protein was buffered in 20 mm Tris (pH 7.4), 50 mm NaCl, and 5% DMSO. BPC was brought to a concentration of 2.5 mm and injected into the sample cell containing 40 μm protein. The data were acquired on a Nano ITC from TA Instruments at 25 °C and fitted with an independent-binding site model using NanoAnalyze software (v3.7.0).

DSF measurements were performed with a Light Cycler 480 II instrument from Roche Applied Science. TTD (UHRF1_121–286_) was buffered at 0.1 mg/ml in 0.1 m HEPES (pH 7.5), 0.15 m NaCl, and 5× Sypro Orange. Sypro Orange was purchased from Invitrogen as a 5,000× stock solution, and it was diluted 1:1,000 to yield a 5× working concentration. Experiments were run in the absence and presence of 3 mm BPC. DSF was carried out by increasing the temperature from 20 to 95 °C at a heating rate of 4 °C/min, and data points were collected at 1 °C intervals. The temperature scan curves were fitted to a Boltzmann sigmoid function, and the *T*_m_ values were obtained from the midpoint of the transition as described previously (47).

## Author contributions

R. S .H. acquired and analyzed NMR data. A. L. interpreted NMR relaxation data, analyzed SAXS data, and performed structural modeling. L. F. acquired and processed SAXS data under the supervision of Y.-X. W. A. I. performed the initial fragment screen with UHRF1-TTD under the supervision of P. J. B. G. S. characterized the interaction of BPC with UHRF1-TTD. D. I. performed the ITC experiments. S. D., L. K., and M. S. O. designed constructs and prepared samples for SAXS, NMR analysis, and binding studies. R. S. H., A. L., and C. H. A. wrote the manuscript. C. H. A. supervised the project.

## Supplementary Material

Supplemental Data

## Footnotes

3 The abbreviations used are:

PHD: plant homeodomain
TTD: tandem tudor domain
PBR: polybasic region
BPC: 4-benzylpiperidine-1-carboximidamide
MDP: molecular dynamics pool
OE: optimal ensemble
PDB: Protein Data Bank
SAXS: small angle X-ray scattering
MD: molecular dynamics
FP: fluorescence polarization
TCEP: tris-(2-carboxyethyl) phosphine hydrochloride
TROSY: transverse relaxation-optimized spectroscopy
ITC: isothermal titration calorimetry
DSF: differential scanning fluorimetry.
