# Supplementary material for: Conformational dynamics of the TTD–PHD histone reader module of the UHRF1 epigenetic regulator reveals multiple histone-binding states, allosteric regulation, and druggability
Source: J Biol Chem. 2017 Oct 26;292(51):20947–59. doi: 10.1074/jbc.M117.799700 (PMC5743070; doi:10.1074/jbc.M117.799700)
Supplement: Supplemental Data [file supp_292_51_20947__index.html]

Conformational dynamics of the TTD-PHD histone reader module of UHRF1 reveals multiple histone binding states, allosteric regulation and druggability — Conformational dynamics of the TTD–PHD histone reader module of the UHRF1 epigenetic regulator reveals multiple histone-binding states, allosteric regulation, and druggability — UHRF1 TTD–PHD dynamics — Supplemental Data 

# Conformational dynamics of the TTD–PHD histone reader module of the UHRF1 epigenetic regulator reveals multiple histone-binding states, allosteric regulation, and druggability

## Supplemental Data

- Supplemental tables and figures (.pdf, 3.0 MB) - Supplemental table and figures
