# Supplementary material for: Conformational dynamics of the TTD–PHD histone reader module of the UHRF1 epigenetic regulator reveals multiple histone-binding states, allosteric regulation, and druggability
Source: J Biol Chem. 2017 Oct 26;292(51):20947–59. doi: 10.1074/jbc.M117.799700 (PMC5743070; doi:10.1074/jbc.M117.799700)
Supplement: Supplemental Data [file 10.1074_M117.799700_jbc.M117.799700-1.pdf]

Supplemental Information

Conformational dynamics of the TTD-PHD histone reader module of UHRF1 reveals multiple histone binding states, allosteric regulation and druggability

R. Scott Houliston, Alexander Lemak, Aman Iqbal, Danton Ivanochko, Shili Duan, Lilia Kaustov, Michelle S. Ong, Lixin Fan, Guillermo Senisterra, Peter J. Brown, Yun-Xing Wang, Cheryl H. Arrowsmith

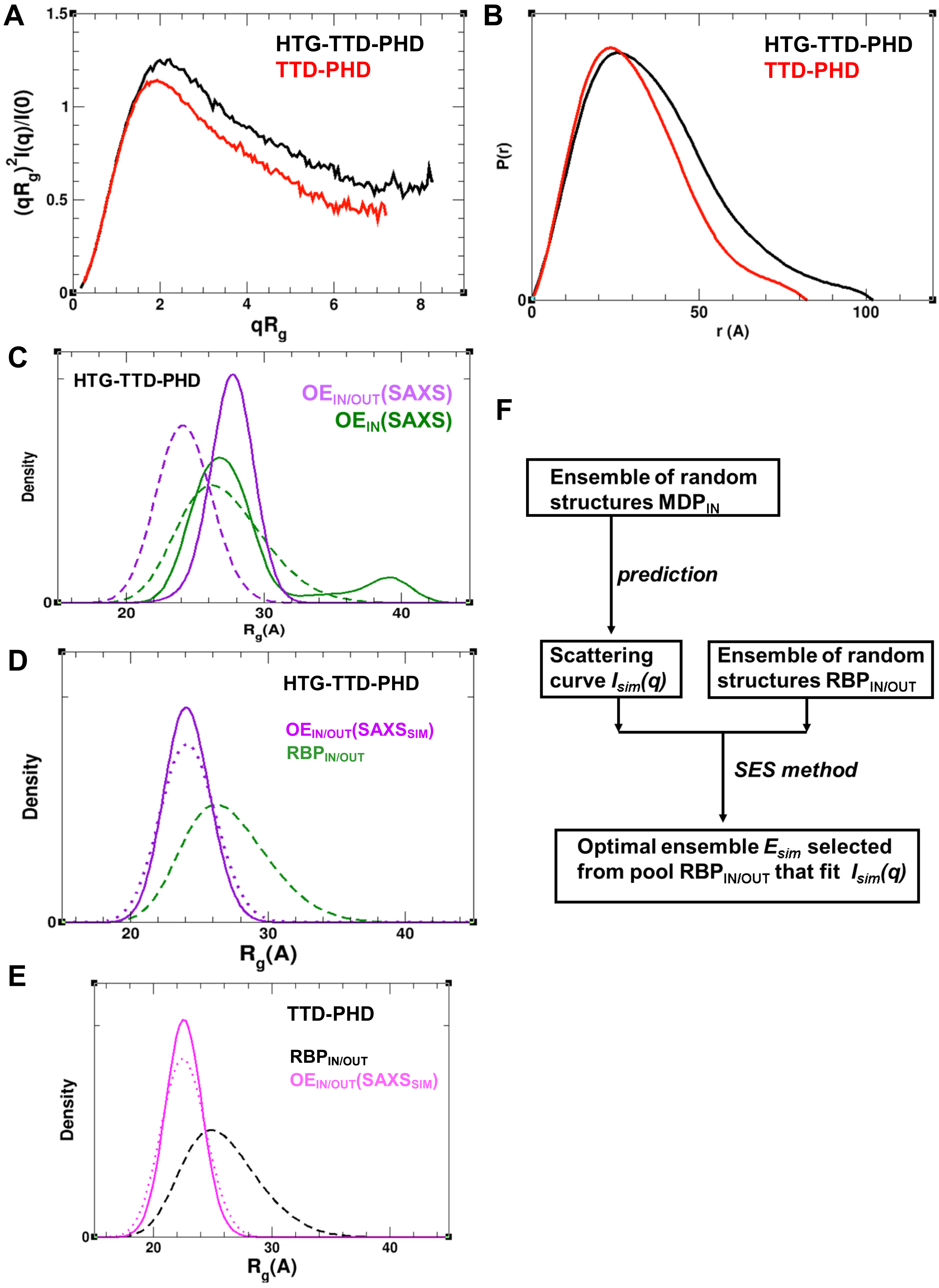
A
HTG-TTD-PHD
TTD-PHD
$(qR_g)^2I(q)/I(0)$
qR_g
B
HTG-TTD-PHD
TTD-PHD
P(r)
r (A)
C
HTG-TTD-PHD
$OE_{IN/OUT}(SAXS)$
$OE_{IN}(SAXS)$
Density
$R_g(A)$
D
HTG-TTD-PHD
$OE_{IN/OUT}(SAXS_{SIM})$
$RBP_{IN/OUT}$
Density
$R_g(A)$
E
TTD-PHD
$RBP_{IN/OUT}$
$OE_{IN/OUT}(SAXS_{SIM})$
Density
$R_g(A)$
F
Ensemble of random structures MDP_{IN}
prediction
Scattering curve $I_{sim}(q)$
Ensemble of random structures $RBP_{IN/OUT}$
SES method
Optimal ensemble E_{sim} selected from pool $RBP_{IN/OUT}$ that fit $I_{sim}(q)$

Figure S1 (related to Fig. 1 and Table 1)

(**A)** Comparison of experimental Kratky plots of TTD-PHD with and without a His tag (HTG). (**B**) Calculated normalized pair-distance distribution function P(r) for TTD-PHD and HTG-TTD-PHD. (**C**) Distributions of R_g for different ensembles of HTG-TTD-PHD. $RBP_{IN/OUT}$ was generated by assuming that the entire linker ($UHRF1_{282-301}$) is disordered, while RBP_{IN} was generated assuming only the 5-residue hinge region of the linker ($UHRF1_{297-301}$) is disordered. The SES method (1) was used to generate the SAXS-fitted OEs, OE_{IN} (SAXS) and $OE_{IN/OUT}$(SAXS), from the pools, RBP_{IN} and $RBP_{IN/OUT}$, respectively. R_g distributions of RBP_{IN} (magenta) and $RBP_{IN/OUT}$ (green, and violet) are shown by dashed lines, while R_g distributions of the optimal ensembles OE_{IN}(SAXS) (violet) and $OE_{IN/OUT}$(SAXS) (green) are shown by solid lines. (**D**) Comparison of R_g distributions for different ensembles of HTG-TTD-PHD. (**E**) Comparison of R_g distributions for different ensembles of TTD-PHD. The R_g distributions for $OE_{IN/OUT}(SAXS_{SIM})$, RBP_{IN}, and $RBP_{IN/OUT}$ ensembles are shown by solid, dotted, and dashed lines, respectively. (**F**) Flow-chart for generating $OE_{IN/OUT}(SAXS_{SIM})$ that is selected from $RBP_{IN/OUT}$ with the SES method using SAXS profile $I_{sim}(q)$ simulated from RBP_{IN}.

A

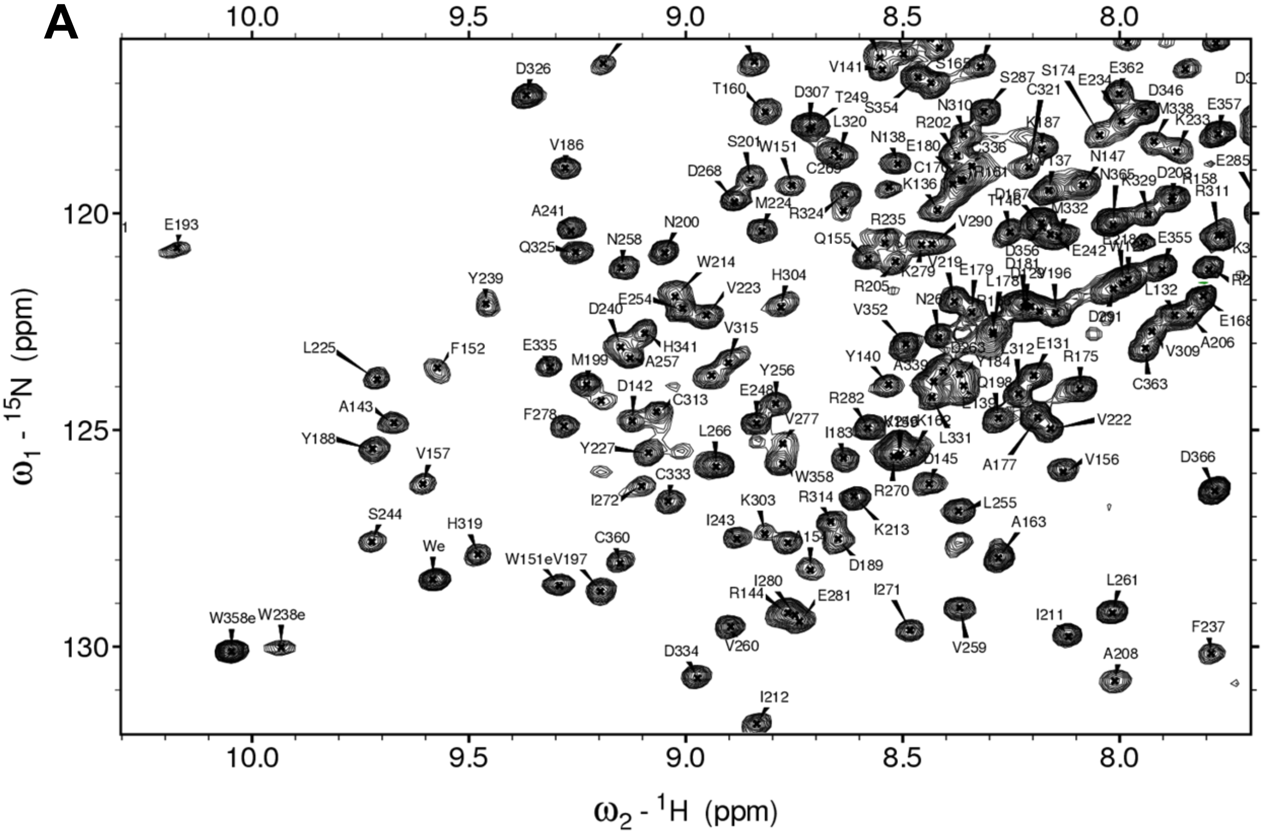

10.0
9.5
9.0
8.5
8.0
120
125
130
ω1 - 15N (ppm)
ω2 - 1H (ppm)
D326
V141
S165
S287
S174
E234
E362
D346
E357
T160
D307
T249
S354
N310
C321
K187
M338
K233
V186
S201
W151
L320
N138
R202
E180
C336
T137
N147
E285
D268
C209
K136
C170
N365
K329
D202
R158
R311
E193
A241
M224
R324
V290
T140
M332
Q325
N258
N200
R235
Q155
D356
E242
E355
Y239
W214
H304
R205
K279
V219
E179
D181
V196
L132
V223
L178
D291
E168
D240
E254
H341
V352
A206
L225
F152
E335
V315
A257
Y140
A339
Y184
L312
E131
R175
V309
C363
M199
Y256
D142
C313
E248
D198
E139
A143
F278
R282
K162
V222
V277
L331
Y188
Y227
L266
I183
D145
A177
V157
W358
R270
V156
D366
I272
C333
S244
H319
I243
K303
R314
K213
L255
A163
We
C360
D189
W151e
V197
I280
R144
E281
I271
L261
W358e
W238e
V260
V259
I211
F237
D334
A208
I212

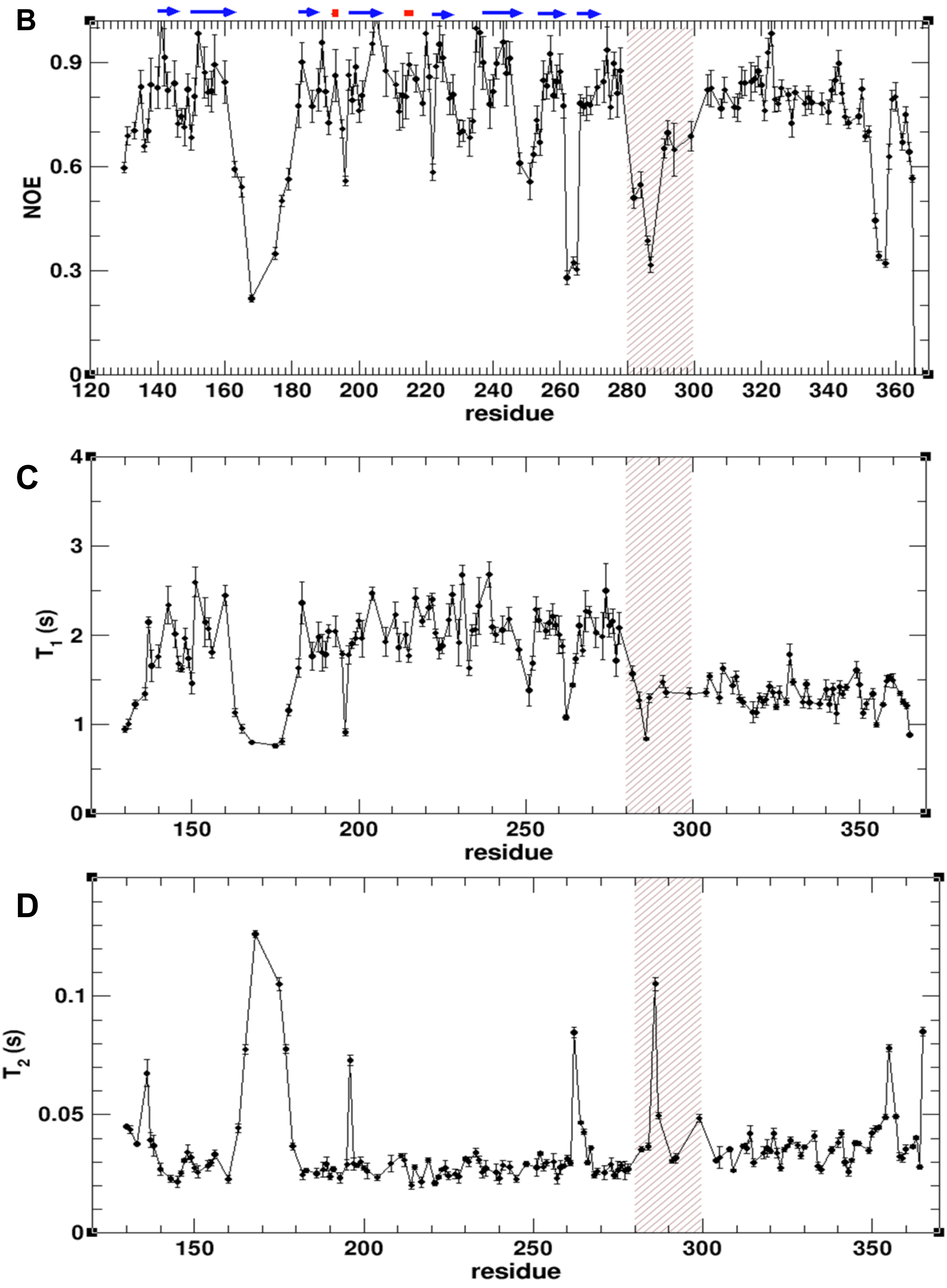
B
NOE
0
0.3
0.6
0.9
120
140
160
180
200
220
240
260
280
300
320
340
360
residue
C
T1 (s)
0
1
2
3
4
150
200
250
300
350
residue
D
T2 (s)
0
0.05
0.1
150
200
250
300
350
residue

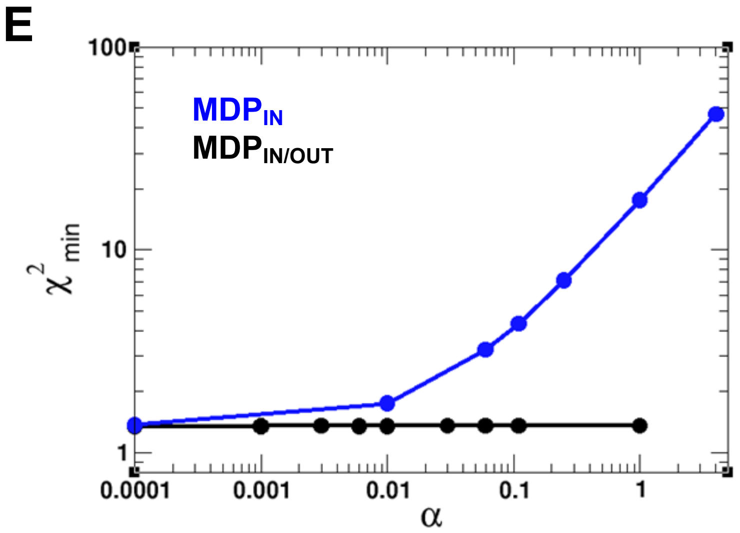

Figure S2 (related to Fig. 2 and Table 2)

NMR data for TTD-PHD and combined NMR and SAXS fitting. (**A**) Portion of a labeled (^{1}H-^{15}N) TROSY spectrum of TTD-PHD (UHRF1$_{126\text{-}366}$). We assigned 203 amide resonances (134 in the TTD, 11 in the linker and 58 in the PHD). (**B**) ^{1}H-^{15}N Heteronuclear NOE, (**C**) ^{15}N T1, and (**D**) ^{15}N T2 values as a function of sequence. The shaded area corresponds to the linker and the top bars indicate secondary structure elements. (**E**) Minimized residual χ^2 (Eq. 1) as function of α.

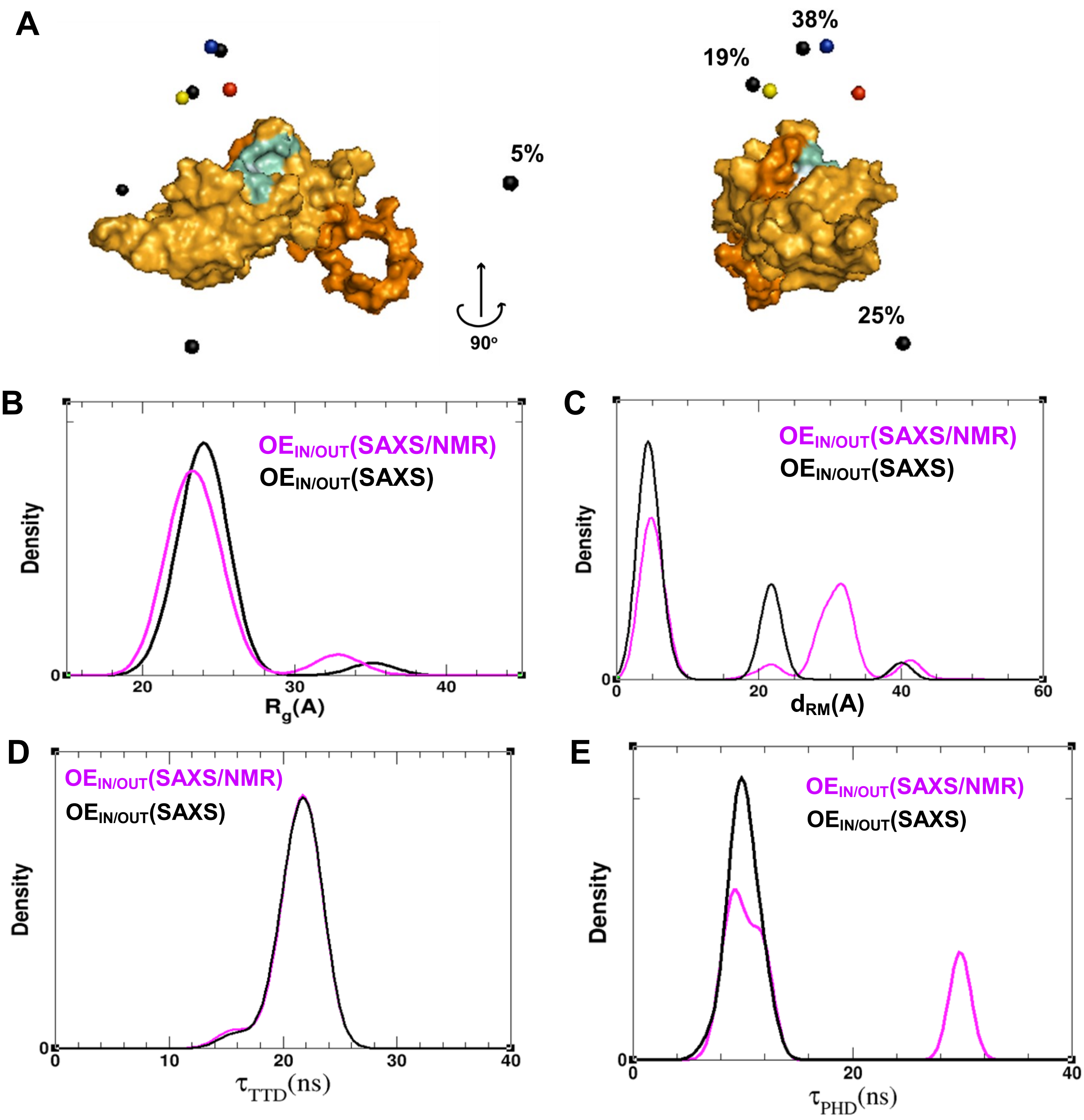

Figure S3 (related to Fig. 3 and Table 3)

Comparison of *$OE_{IN/OUT}$(SAXS)* ***and*** *$OE_{IN/OUT}$(SAXS/NMR)* (**A**) The position of PHD centers-of-mass in the most populated conformers (based on weighted %) from $OE_{IN/OUT}$(SAXS) (black spheres) superimposed with the TTD (as a surface representation). Residues that bind to the H3 peptide are displayed in cyan. The red sphere shows the PHD center-of-mass in H3-bound TTD-PHD (PDB:3ASK) (2), and the yellow sphere shows the PHD center-of-mass in apo TTD-PHD of UHRF2 (PDB:4TVR) (**B**) R_g-distributions of $OE_{IN/OUT}$ (SAXS/NMR) and $OE_{IN/OUT}$(SAXS). (**C**) d_{RM} distributions of $OE_{IN/OUT}$(SAXS/NMR) and $OE_{IN/OUT}$(SAXS). Distribution of the HYCUD-predicted (3, 4) correlation times of the TTD (**D**) and PHD (**E**) from structures in $OE_{IN/OUT}$(SAXS) and $OE_{IN/OUT}$(SAXS/NMR).

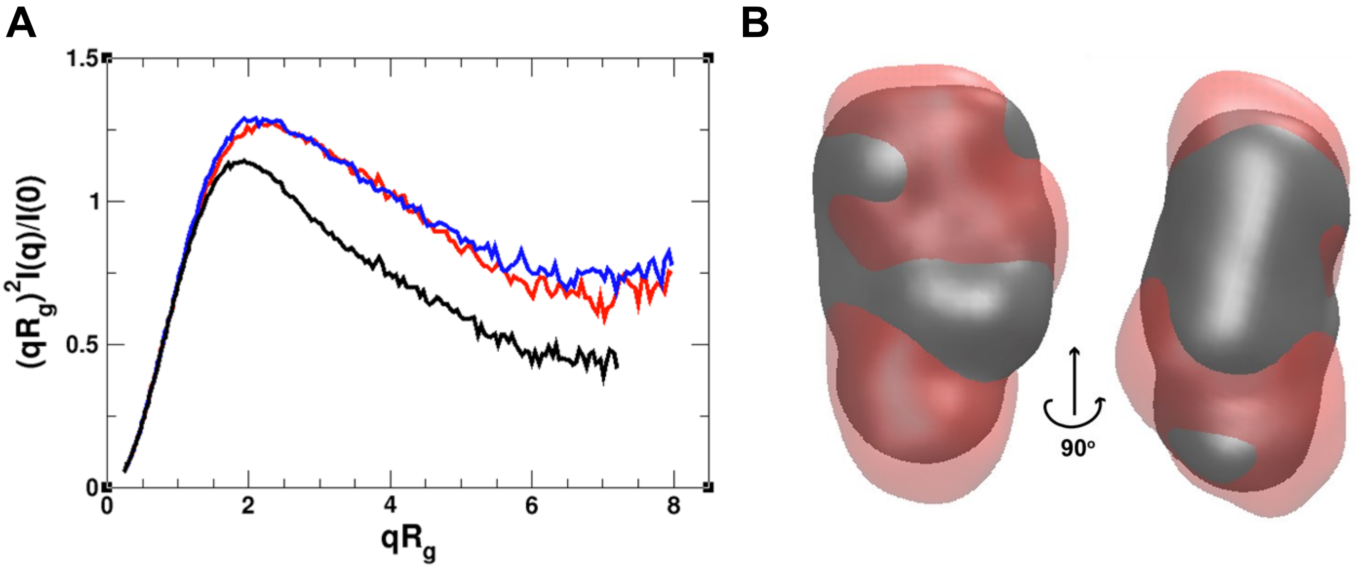

Figure S4 (related to Fig. 4)

Comparison of SAXS data for TTD-PHD and BPC-bound TTD-PHD. (**A**) Experimental R_g-based Kratky plots for apo (black) and BPC-bound TTD-PHD (at 2 mM BPC, 4% DMSO - blue; and 4 mM BPC, 4% DMSO, red). (**B**) Comparison of average *ab initio* SAXS-predicted molecular envelopes of apo (black) and BPC-bound TTD-PHD (red).

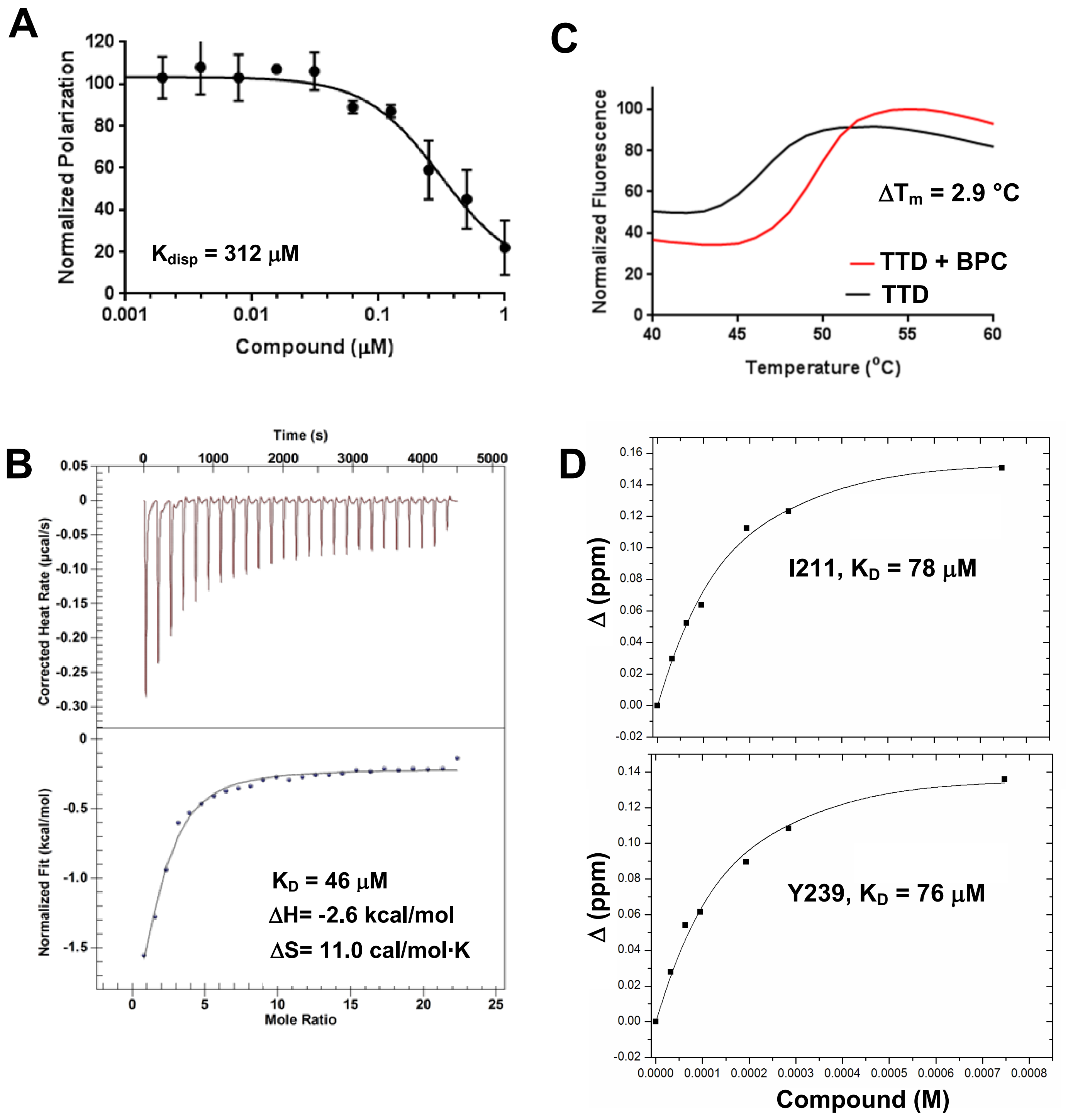
A
Normalized Polarization
Compound (μM)
Kdisp = 312 μM
C
Normalized Fluorescence
Temperature (°C)
ΔTm = 2.9 °C
TTD + BPC
TTD
B
Time (s)
Corrected Heat Rate (μcal/s)
Normalized Fit (kcal/mol)
Mole Ratio
KD = 46 μM
ΔH= -2.6 kcal/mol
ΔS= 11.0 cal/mol·K
D
Δ (ppm)
I211, KD = 78 μM
Y239, KD = 76 μM
Compound (M)

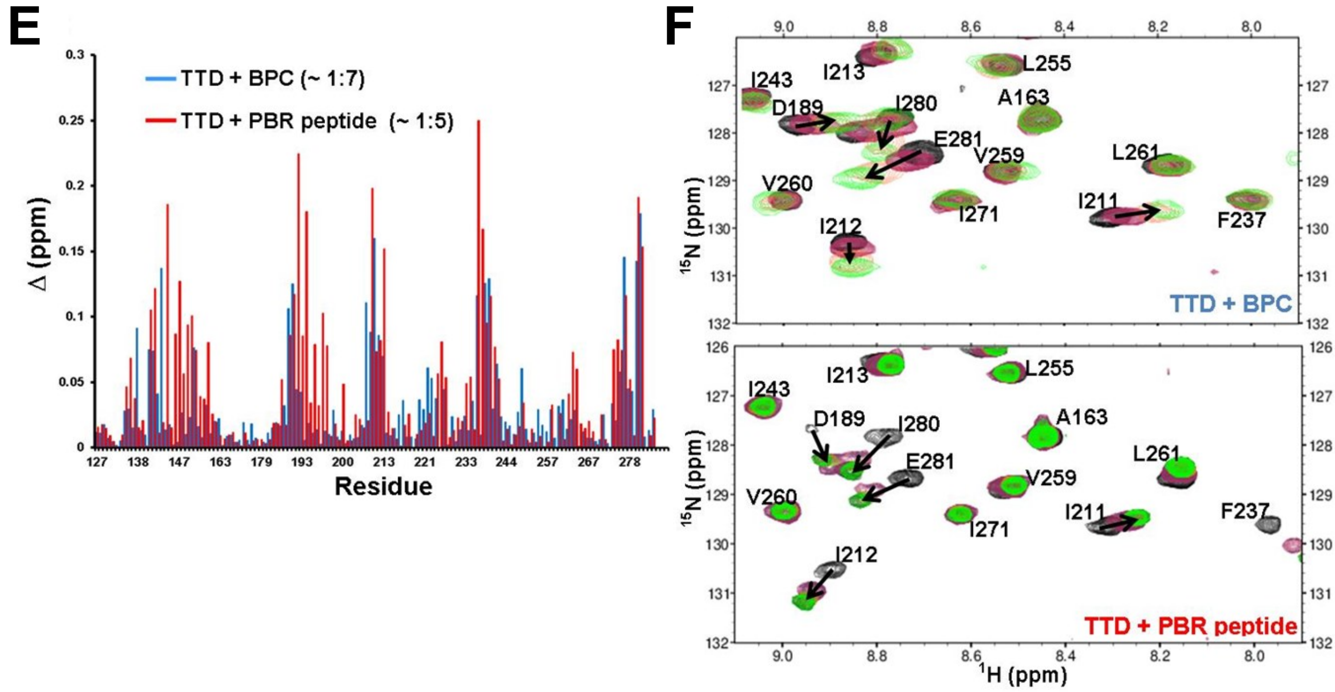

Figure S5 (related to Fig. 4)

Binding of BPC in the groove of TTD. TTD binding to BPC as seen by (**A**) FP displacement of a FITC-labeled (at the N-terminus) $H3K9me3_{(1\text{-}25)}$ peptide, (**B**) ITC, and (**C**) DSF. (**D**) K_D estimates of BPC binding based on I211 and Y239 amide peak movement (where $\Delta(ppm) = ((\Delta\delta HN)^2 + (\Delta\delta N/6.5)^2)^{1/2}$) in ($^1H$-$^{15}N$) HSQC spectra when the TTD is titrated with BPC. (**E**) A histogram shows peak movement in HSQC spectra as a function of TTD sequence resulting from BPC (blue) or PBR peptide (red) binding. (**F**) HSQC overlays shows amide peak movement of TTD resonances at increasing BPC (top spectrum - from 1:1 to 7:1 fragment:protein), and PBR peptide ratios (bottom spectrum - from 1:1 to 5:1 peptide:protein). The protein concentration was ~250 μM for all NMR experiments, and the DMSO concentration was 5% for NMR titrations with BPC.

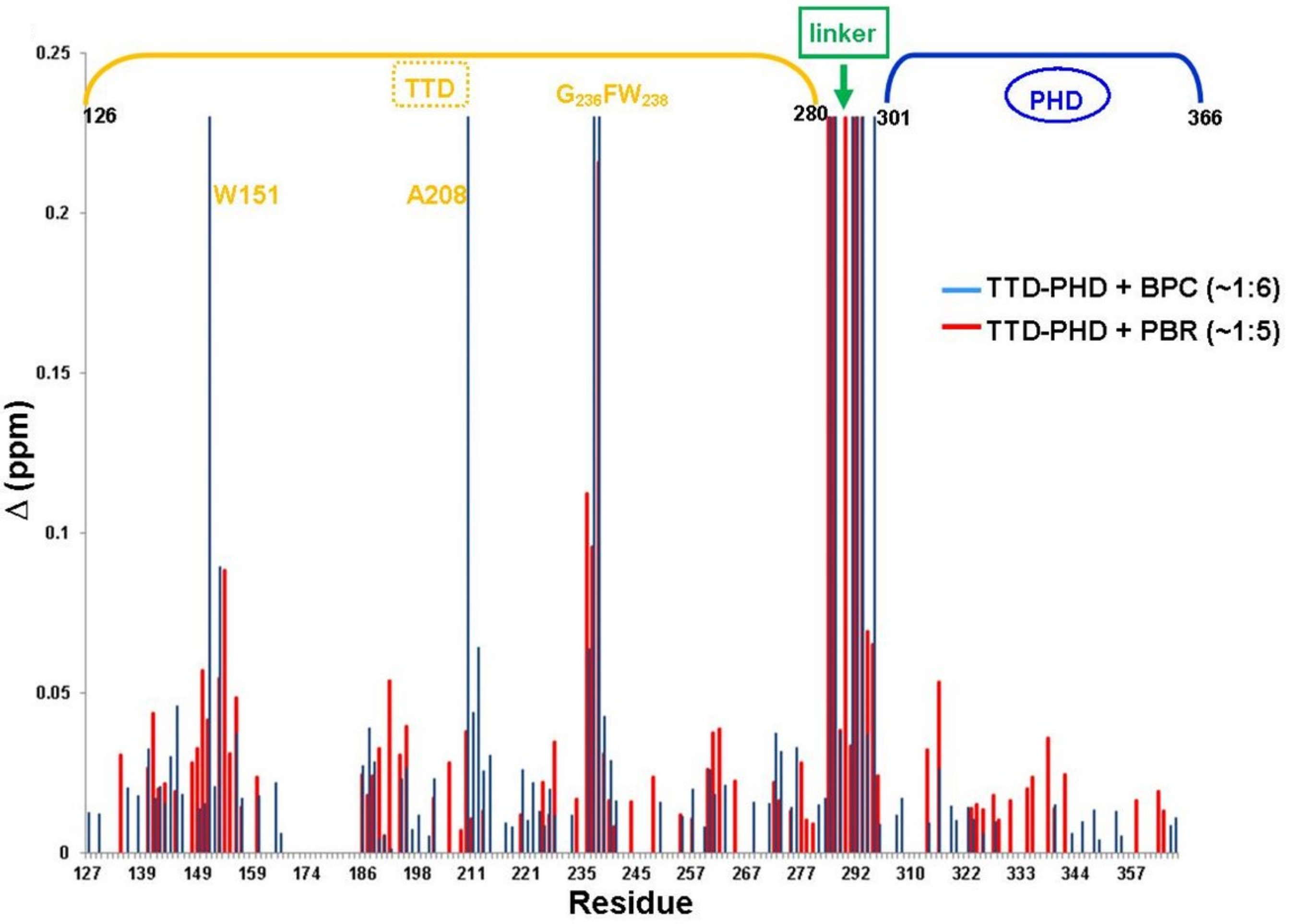

Figure S6 (related to Fig. 4)

Histogram showing side-by-side comparison of peak movement/broadening in (^1H-^{15}N) TROSY spectra when TTD-PHD is titrated with BPC (blue) or PBR peptide (red). Broadened resonances are assigned a value of 0.23. The protein concentration was ~ 250 μM, and the DMSO concentration was kept at 5% for BPC titrations.

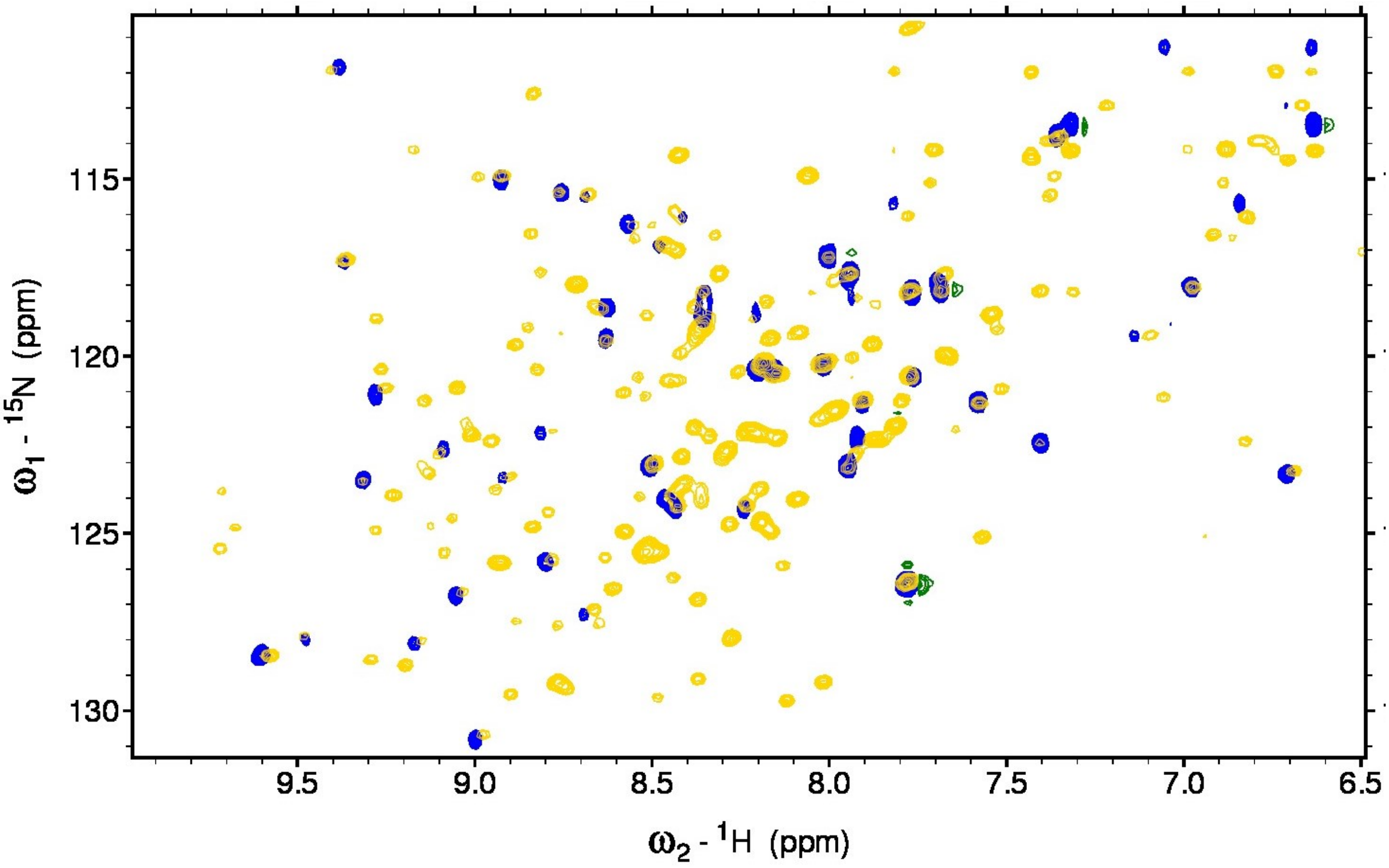

Figure S7

Overlay of an (^{1}H-^{15}N) HSQC spectrum of UHRF1 PHD (blue), with a TROSY spectrum of TTD-PHD (yellow). Only slight deviations in peak positions are observed for this domain in isolation *vs*. its presence within the reader module, indicating that there are minimal (if any) contacts between the PHD and the linker and/or TTD.

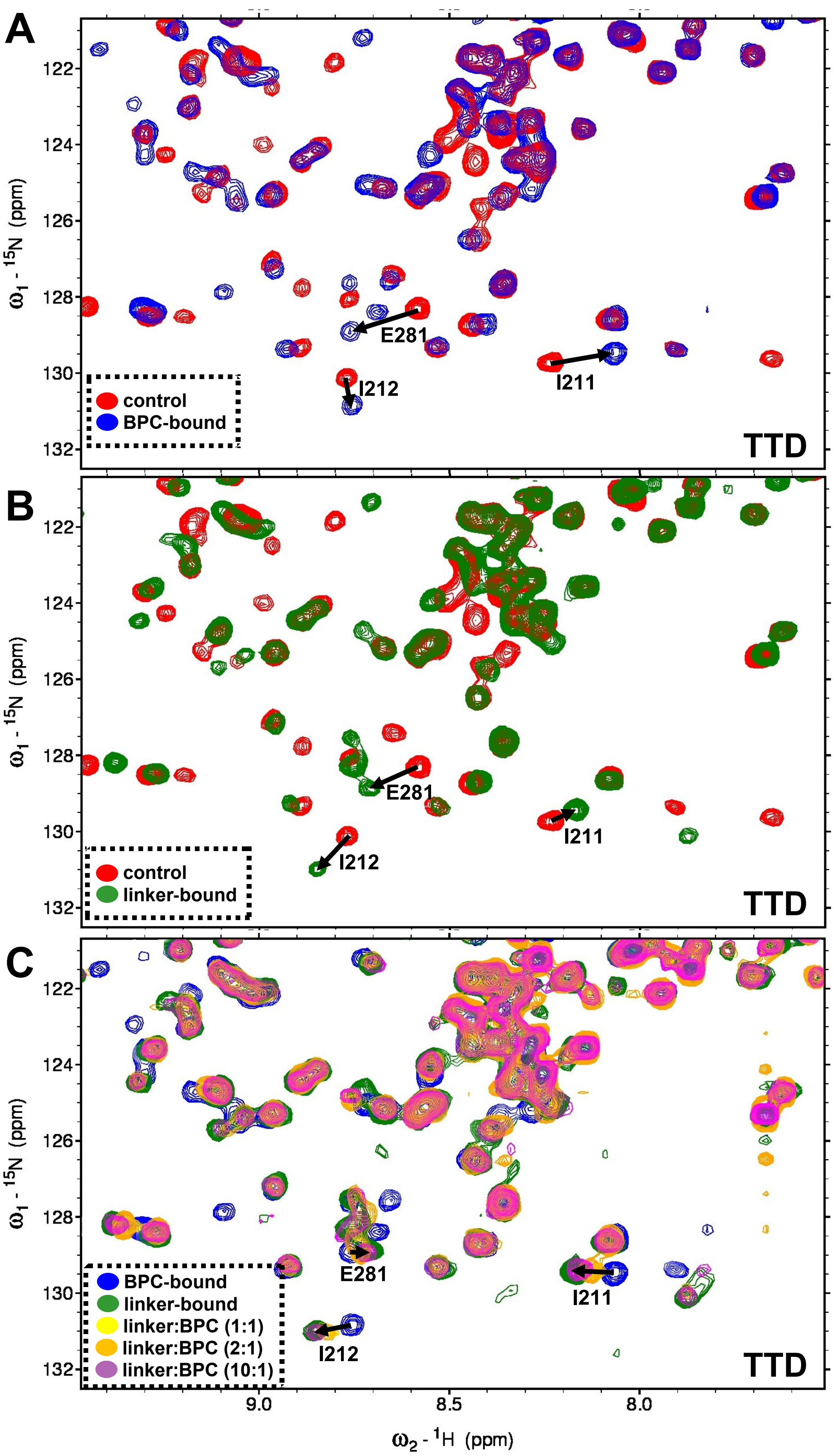

A
B
C
control
BPC-bound
linker-bound
linker:BPC (1:1)
linker:BPC (2:1)
linker:BPC (10:1)
E281
I212
I211
TTD
ω_1 - ^{15}N (ppm)
ω_2 - ^{1}H (ppm)
122
124
126
128
130
132
9.0
8.5
8.0

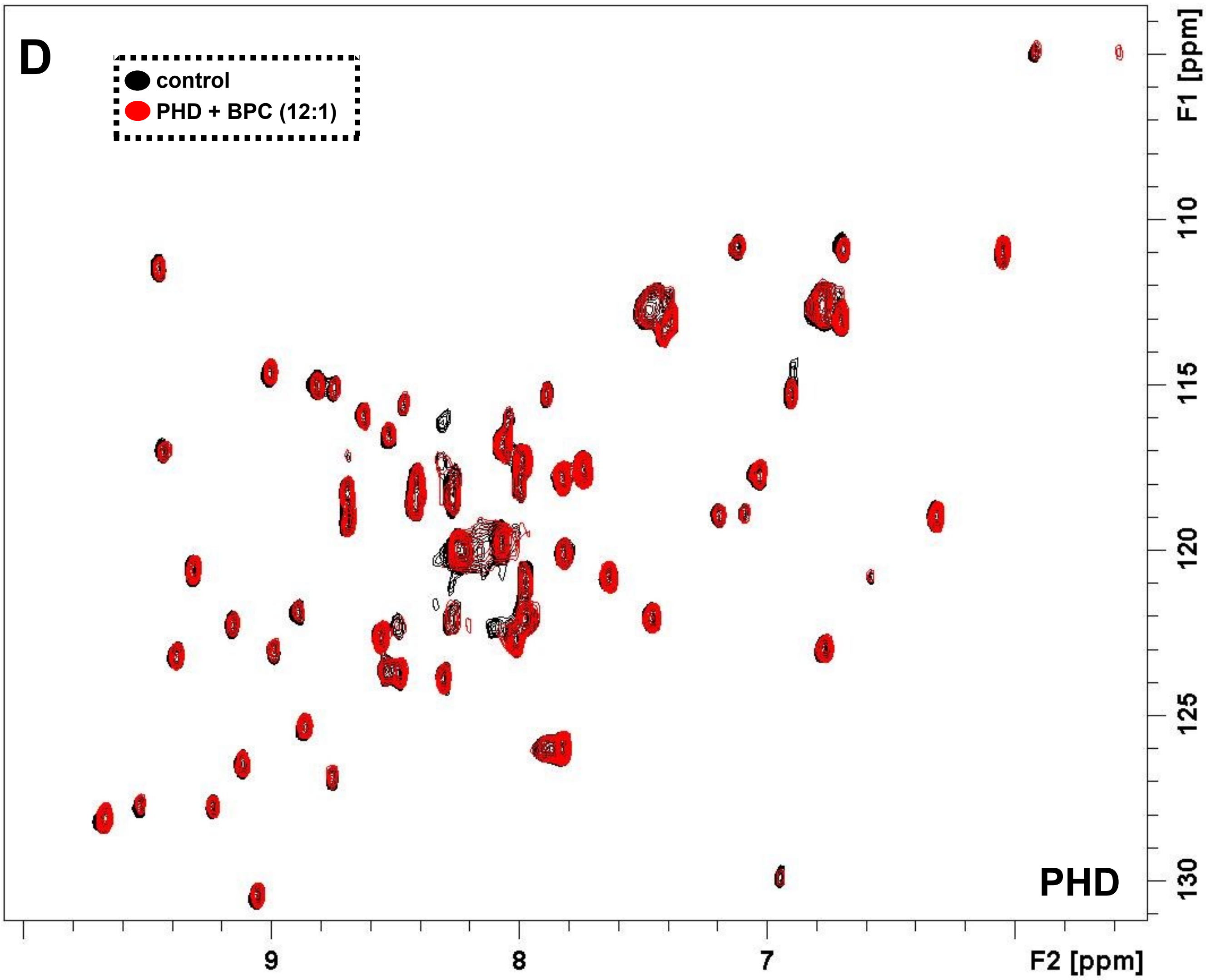

Figure S8

(**A-C**) BPC and linker peptide (corresponding to $UHRF1_{286\text{-}300}$) compete directly for the TTD groove as illustrated by the perturbation of I211, I212 and E281 resonances in HSQC spectra of the TTD in a competition assay. Movement of TTD resonances in the presence of (**A**) BPC (2.4 mM, 5% DMSO) and (**B**) linker peptide (3.0 mM, 5% DMSO). (**C**) At increasing peptide:BPC ratios, the peaks transition from a BPC-bound pattern to a linker-bound pattern, demonstrating direct competition for binding to the groove. (**D**) PHD resonances are unaffected by the presence of BPC (3 mM, 5% DMSO).

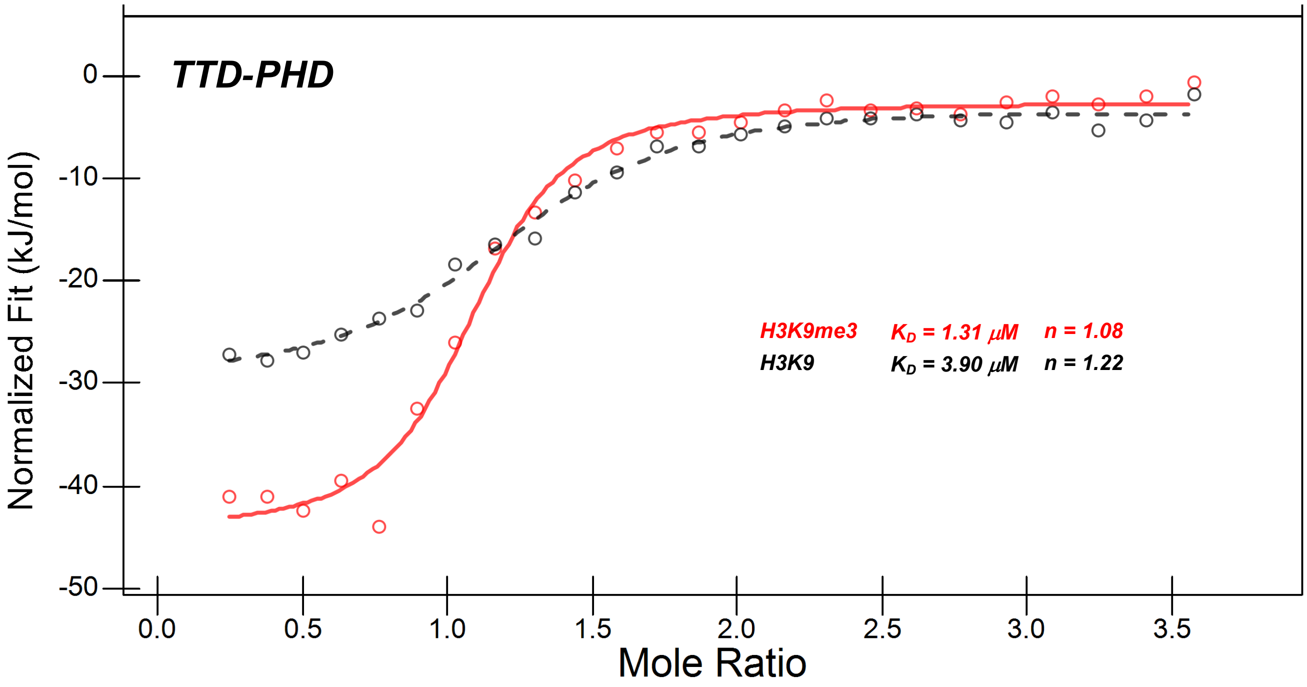

Figure S9

ITC curves showing TTD-PHD binding to methylated (red) and unmethylated (black) H3 peptide. Methylated peptide binding is mediated by the TTD and PHD in a cooperative manner (2). Only the PHD can interact with unmethylated H3 peptide. BPC or PBR binding in the TTD groove similarly disrupts cooperative H3 binding by the histone reader.

Table S1 (related to Fig. 1, S1, S4): SAXS parameters for the TTD-PHD module

**SAXS**	**HTG-TTD-PHD**[a]	**TTD-PHD/BPC**[b]
I(0)[c]	0.0908	0.0237
R_g (Å)[d]	27.7	26.6
R_g (Å) real[e]	28.9	27.1
D_{max}[f] (Å)	100	94
V_c[g]	319.7	280.5
M_w[h]	29.9 (30.0)	23.6 (28.1)
NSD[i]	0.67 ± 0.04	0.80 ± 0.03

[a] $UHRF1_{126-366}$ with a His tag (18 aa)

[b] $UHRF1_{126-366}$ in complex with BPC (2 mM, 4% DMSO)

[c] Intensity at q=0

[d] R_g calculated using Guinier fit

[e] R_g calculated using GNOM (5)

[f] Maximum distance between atoms from GNOM

[g] Volume of correlation (6)

[h] M_w estimated from SAXS data using V_c (6). The M_w expected from sequence is shown in parentheses

[i] Normalized spatial discrepancy. The values are the average and standard deviation from fifteen runs of DAMMIF (7)

Table S2 (related to Fig. 3): Average ^{15}N relaxation parameters for TTD-PHD at 800MHz[a].

	T_1^b (s)	T_2^b (s)	NOE[c]	T_1/T_2^b
**TTD**	2.01 ± 0.20	0.028 ± 0.004	0.73 ± 0.06	73.3 ± 12.6
**PHD**	1.37 ± 0.15	0.034 ± 0.005	0.75 ± 0.06	40.8 ± 7.9
**TTD-PHD**	1.75 ± 0.37	0.031 ± 0.005	0.73 ± 0.06	60.0 ± 19.4

[a] Averaging is performed over residues in the regular secondary structural elements
[b] Errors are propagated fitting errors
[c] Errors are standard deviations of averaging

Table S3 (related to Fig. 3): Rotational correlation time τ_c (*ns*) of the TTD and PHD predicted from different TTD-PHD models.

Model	TTD	PHD
Individual[a]	15.6	5.5
Rigidly attached[a]	26.6	26.6
$MDP_{IN/OUT}$[b]	22.0 ± 4.7	11.9 ± 4.9
MDP_{IN}[c]	20.6 ± 1.9	9.7 ± 1.0

[a] Results of hydrodynamic calculations for the TTD and PHD (both individually and rigidly attached) were estimated using the crystal structure of TTD-PHD (PDB:3ASK)[2] and the program HYDRONMR (8) with parameter 'a' set to 2.9

[b] The average τ_c values of the TTD and PHD predicted from $MDP_{IN/OUT}$ using the HYCUD method (4)

[c] The average τ_c values of the TTD and PHD predicted from MDP_{IN} using the HYCUD method (4)

References

1. Berlin, K., Castañeda, C. A., Schneidman-Duhovny, D., Sali, A., Nava-Tudela, A., and Fushman, D. (2013) Recovering a representative conformational ensemble from underdetermined macromolecular structural data. *J. Am. Chem. Soc.* **135**, 16595–16609

2. Arita, K., Isogai, S., Oda, T., Unoki, M., Sugita, K., Sekiyama, N., Kuwata, K., Hamamoto, R., Tochio, H., Sato, M., Ariyoshi, M., and Shirakawa, M. (2012) Recognition of modification status on a histone H3 tail by linked histone reader modules of the epigenetic regulator UHRF1. *Proc. Natl. Acad. Sci. U. S. A.* **109**, 12950–12955

3. Rezaei-Ghaleh, N., Klama, F., Munari, F., and Zweckstetter, M. (2013) Predicting the rotational tumbling of dynamic multidomain proteins and supramolecular complexes. *Angew. Chem. Int. Ed Engl.* **52**, 11410–11414

4. Rezaei-Ghaleh, N., Klama, F., Munari, F., and Zweckstetter, M. (2015) HYCUD: a computational tool for prediction of effective rotational correlation time in flexible proteins. *Bioinforma. Oxf. Engl.* **31**, 1319–1321

5. Feigin, L. A., and Svergun, D. I. (1987) Structure analysis by small-angle X-ray and neutron scattering, Plenum Press

6. Rambo, R. P., and Tainer, J. A. (2013) Accurate assessment of mass, models and resolution by small-angle scattering. *Nature*. **496**, 477–481

7. Franke, D., and Svergun, D. I. (2009) DAMMIF, a program for rapid ab-initio shape determination in small-angle scattering. *J. Appl. Crystallogr.* **42**, 342–346

8. García de la Torre, J., Huertas, M. L., and Carrasco, B. (2000) HYDRONMR: prediction of NMR relaxation of globular proteins from atomic-level structures and hydrodynamic calculations. *J. Magn. Reson.* **147**, 138–146
